# Synthesis, Characterization, and Corrosion Inhibition Properties of a Novel Quaternary Ammonium Salt Containing Dual-Imidazoline Rings for N80 Carbon Steel Under CO_2_ Corrosion Conditions

**DOI:** 10.3390/ma19101934

**Published:** 2026-05-08

**Authors:** Xiaoping Qin, Xi Chen, Peng Tang, Cuixia Li, Yangyang Yu, Wei Liu, Guanglin Zhou, Wenzhong Tian, Guangliang Lu, Song Qing, Haiyang Tian

**Affiliations:** 1 School of Chemical Engineering, Sichuan University of Science and Engineering, Zigong 643000, Chinachenxi_2413@163.com (X.C.);; 2 Jidong Oilfield Branch Company, PetroChina Company Limited, Tangshan 063002, China; 3 Southwest Oil and Gas Branch, China Petroleum and Chemical Corporation, Chengdu 610095, China

**Keywords:** CO_2_ corrosion, imidazoline quaternary ammonium salt, dual-imidazoline ring, corrosion inhibition mechanism, N80 carbon steel

## Abstract

A novel dual-imidazoline ring quaternary ammonium salt corrosion inhibitor (TN-IM) was rationally synthesized via a three-step sequential reaction, using hydroxyethyl ethylenediamine and tetradecanedioic acid as starting materials, with benzyl chloride as the quaternizing reagent. The synthetic process involved amidation at 160 °C for 4 h, cyclization at 220 °C for 3 h, and quaternization at 70 °C for 3 h, respectively. Fourier transform infrared spectroscopy and proton nuclear magnetic resonance were employed to characterize the chemical structure of TN-IM, confirming its successful synthesis. The corrosion inhibition performance of TN-IM was evaluated by the static weight loss method and electrochemical measurements, while the corrosion products and surface morphology of N80 carbon steel were analyzed via energy-dispersive X-ray spectroscopy and scanning electron microscopy. Static weight loss tests conducted in 3.5 wt% of a NaCl solution saturated with 0.6 MPa CO_2_ at 60 °C for 24 h revealed that TN-IM at a concentration of 0.15 mmol/L exhibited a corrosion inhibition efficiency 1.86% higher than that of a single-imidazoline ring quaternary ammonium salt inhibitor. Potentiodynamic polarization measurements demonstrated that TN-IM functions as a mixed-type corrosion inhibitor, with a predominant inhibitory effect on the anodic reaction on N80 steel. Electrochemical impedance spectroscopy results indicated that TN-IM molecules can adsorb onto the active sites of the N80 surface, thereby retarding the corrosion process by suppressing the charge transfer step in the electrochemical corrosion reaction. This study establishes a new paradigm for the synthesis of high-efficiency imidazoline-based CO_2_ corrosion inhibitors with multiple adsorption sites, holding significant implications for corrosion control in harsh industrial environments.

## 1. Introduction

Imidazoline-based corrosion inhibitors are widely applied in CO_2_-enhanced oil recovery and gas production industries due to their strong adsorption on metal surfaces, excellent corrosion inhibition performance, and low toxicity [1,2,3,4,5]. The electronegative nitrogen atoms and unsaturated –C=N– double bonds in the imidazoline ring serve as the core adsorption sites, enabling physical and chemical adsorption on metal surfaces via lone-pair electron coordination and hydrogen bonding [1]. However, the harsh service conditions of CO_2_-containing oil and gas wells (e.g., high temperature, high pressure, high water/gas production, and severe fluid scouring) pose severe challenges to traditional single-imidazoline ring corrosion inhibitors [6,7,8,9,10]. Such inhibitors, possessing only one adsorption site, typically develop a loose and discontinuous film upon adsorption onto metal surfaces, which fails to provide long-term and effective corrosion protection for downhole metal equipment and pipeline steel such as N80 steel. The imino group on the imidazoline ring can provide lone-pair electrons, and its H atoms can participate in hydrogen bond formation for physical adsorption, serving as the core adsorption site of imidazoline-based corrosion inhibitors and endowing them with good corrosion inhibition effects [11,12,13]. Therefore, dual-imidazoline ring corrosion inhibitors, with two imidazoline rings and their corresponding imino polar groups in the molecular structure, exhibit a synergistic bicyclic framework that greatly amplifies the molecule’s effective adsorption sites. Moreover, the molecular skeleton (e.g., the long chain of dimer acid) connecting the two imidazoline rings forms a dense hydrophobic physical barrier on the metal surface. This barrier not only blocks the infiltration of corrosive media (e.g., H^+^, HCO_3_^−^, and Cl^−^) but also enhances the stability of the adsorption film. Thus, it facilitates the formation of a denser, more continuous, and more stable film [14,15,16,17,18,19], enabling bis-imidazoline ring corrosion inhibitors to effectively resist the harsh environments of high temperature, high pressure, high-velocity scouring, and high gas–liquid production in coalbed methane wells, significantly improving their protection effect on metal equipment and meeting the engineering requirements for long-term and efficient anti-corrosion.

Currently, the dominant synthetic strategies for dual-imidazoline ring corrosion inhibitors encompass the dibasic acid skeleton method, polyamine method, and group substitution method [20,21,22]. Among these approaches, the dibasic acid skeleton method is the most widely adopted, as dibasic acids can serve as molecular linkers to bridge two imidazoline rings through sequential amidation and cyclization reactions. Shen et al. [23] synthesized a symmetric dual-imidazoline ring quaternary ammonium salt featuring strong synergistic adsorption, dense film formation, and good hydrophobicity, but its complex and bulky molecular structure results in unstable adsorption under high-velocity natural gas scouring conditions in coalbed methane reservoirs. The polyamine-based synthetic route requires strict control over reaction temperature and reactant molar ratios [24], while the group substitution method is plagued by poor long-term stability of the resulting products under high-temperature and strong-acid environments [25].

Therefore, tetradecanedioic acid was used as a flexible skeleton to connect two hydroxyethyl ethylenediamine-derived imidazoline rings, yielding a novel dual-imidazoline ring quaternary ammonium salt (TN-IM) in this work. Subsequently, chlorobenzyl quaternization was performed. Tetradecanedioic acid was selected as the linker due to its moderate carbon chain length, which balances the adsorption capacity and film-forming stability of the inhibitor molecule, avoiding the steric hindrance caused by an overly long carbon chain and the insufficient hydrophobic barrier of an overly short one. The chemical structure of TN-IM was confirmed by Fourier transform infrared spectroscopy (FT-IR) and proton nuclear magnetic resonance (^1^H-NMR). The corrosion inhibition performance of TN-IM for N80 steel was systematically investigated by the static weight loss method under different experimental conditions (inhibitor concentration, CO_2_ pressure, temperature, corrosion time, and NaCl concentration) and compared with that of a commercial single-imidazoline ring quaternary ammonium salt (STD-MC). The corrosion inhibition mechanism of TN-IM was elucidated using electrochemical measurement methods, including potentiodynamic polarization (PDP) and electrochemical impedance spectroscopy (EIS), while scanning electron microscopy (SEM) and energy-dispersive spectroscopy (EDS) were used to characterize the surface morphology and composition of N80 steel before and after corrosion.

## 2. Experimental Methods

### 2.1. Materials and Apparatus

Hydroxyethyl ethylenediamine, benzyl chloride, absolute ethanol, xylene, tetradecanedioic acid, and acetone were all analytical-grade reagents purchased from Chengdu Kelong Chemical Co., Ltd. (Chengdu, China) and used without further purification. N80 carbon steel coupons with dimensions of 50 × 25 × 2 mm were used as the test material, and their elemental composition (wt%) is listed in Table 1.

The experimental apparatus included a constant-temperature heating mantle (SXKW, Beijing Yongguangming Instrument Co., Ltd., Beijing, China), an electrochemical potentiostat (CHI660D, Shanghai Chenhua Instrument Co., Ltd., Shanghai, China), an electronic constant speed stirrer (JHS-1, Hangzhou Instrument Motor Co., Ltd., Hangzhou, China), a Fourier transform infrared spectrometer (FTIR-650, Bruker, Berlin, Germany), a scanning electron microscope (VEGA 3SBU, NETZSCH, Waldkraiburg, Germany), a nuclear magnetic resonance spectrometer (Bruker-AC-E200, Bruker, Fällanden, Switzerland), a drying oven (WGZ, Shanghai Keheng Industrial Development Co., Ltd., Shanghai, China), and a high-temperature and high-pressure autoclave (Chengdu Yankin Technology Co., Ltd., Chengdu, China).

### 2.2. Synthesis of TN-IM and STD-MC

A 250 mL four-necked flask fitted with a mechanical stirrer, water separator, thermometer, and nitrogen inlet was flushed with N_2_ for 30 min to eliminate air. Then, 0.1 mol of tetradecanedioic acid was added to the flask and heated to 70 °C to dissolve it completely. Subsequently, 10 mL of deionized water, 0.22 mol of hydroxyethyl ethylenediamine, and 20 mL of xylene were added, and the mixture was heated to 160 °C at a controlled rate for a 4 h amidation reaction, with the generated water continuously removed by the water separator. The temperature was then raised to 220 °C for a 3 h cyclization reaction, where the two carboxyl groups of tetradecanedioic acid reacted with the amino groups of two hydroxyethyl ethylenediamine molecules to form the dual-imidazoline ring intermediate, with the water produced removed by azeotropic distillation with xylene.

**Figure 1:**
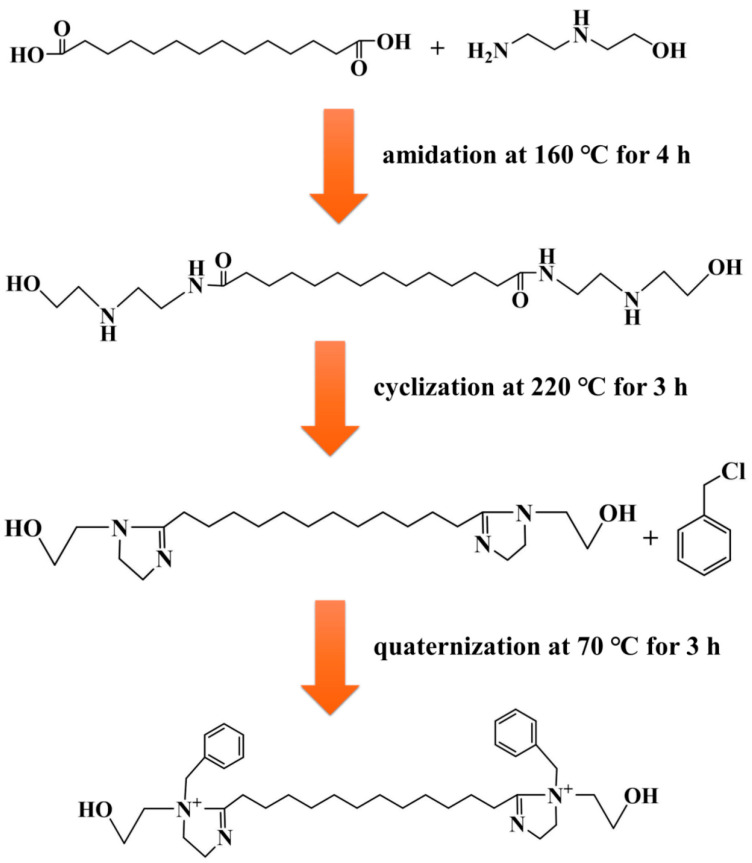
Synthetic route of TN-IM.

After the cyclization reaction, the reaction mixture was cooled to 140 °C, N_2_ purging was ceased, and vacuum distillation was carried out for 60 min to eliminate unreacted reagents and residual by-products. The reaction mixture was further cooled to 70 °C, N_2_ purging was restarted, and 30 mL of absolute ethanol was added as the solvent. Thereafter, a total of 0.2 mol of benzyl chloride was slowly added dropwise into the four-necked flask using a constant-pressure dropping funnel for the quaternization reaction, which proceeded at 70 °C for 3 h. Upon completion of the reaction, the product was subjected to rotary evaporation at 60 °C for 1 h to remove ethanol, recrystallized three times with acetone, and dried in a vacuum drying oven at 40 °C for 4 h to yield pure TN-IM with a yield of about 80% and a purity of 95%. The synthetic route of TN-IM is shown in Figure 1.

For comparison, STD-MC was synthesized using the same procedure with stearic acid (0.1 mol), diethylenetriamine (0.11 mol), and benzyl chloride (0.1 mol) as the feedstocks. The stearic acid with a single carboxyl group reacts with diethylenetriamine to form a single-imidazoline ring intermediate, which is then quaternized with benzyl chloride to obtain STD-MC.

### 2.3. Structural Characterization

FTIR characterization of TN-IM was carried out on an FTIR-650 spectrometer (Bruker, Berlin, Germany) with a spectral range of 4000~450 cm^−1^ by means of the KBr pellet method. For ^1^H-NMR analysis, 1~2 mg of purified TN-IM was placed into an NMR tube and fully dissolved in 0.55 mL of deuterated water (D_2_O). The ^1^H-NMR spectrum of TN-IM was acquired on a Bruker-AC-E200 spectrometer (Bruker, Fällanden, Switzerland) operating at 200 MHz under ambient temperature conditions.

### 2.4. Evaluation of Corrosion Inhibition Performance of TN-IM and STD-MC

The N80 steel coupons were pre-treated by grinding with 800~2000-mesh sandpapers in sequence, followed by wiping with absorbent cotton to remove surface iron powder, degreasing with acetone for 5 min, dehydrating with absolute ethanol for 5 min, and drying with cold air. The pre-treated coupons were weighed accurately (*m*, g), and their dimensions were measured with a vernier caliper to calculate the total surface area (*S*, cm^2^).

The static weight loss tests were carried out in a high-temperature and high-pressure autoclave. The pre-treated coupons were suspended in the autoclave containing 3.5 wt% NaCl solution with different concentrations of TN-IM or STD-MC or without adding TN-IM or STD-MC. Specifically, the concentrations of TN-IM and STD-MC were 0.03 mmol/L, 0.06 mmol/L, 0.09 mmol/L, 0.12 mmol/L, 0.15 mmol/L, 0.18 mmol/L, and 0.21 mmol/L. The autoclave was sealed, purged with CO_2_ for 30 min to remove air, and then charged with CO_2_ to the desired pressure. The autoclave was placed in a constant-temperature oven and maintained at the set temperature for the specified corrosion time. After the test, the coupons were taken out, immersed in a pickling solution (10 vol% HCl + 0.5~1.0 wt% hexamethylenetetramine) for 5 min to remove corrosion products, rinsed with deionized water, dehydrated with absolute ethanol, dried with cold air, and weighed accurately (*m*_1_, g). The corrosion rate ($r_{c}$, mm/a) and corrosion inhibition efficiency (IE, $\eta_{1}$, %) were calculated using Equation (1) and Equation (2) [26], respectively.

(1) $$r_{c}=\frac{8.76\times 10^{4}\times (m-m_{1})}{S\times t\times \rho }$$

(2) $$\eta_{1}=\frac{\Delta m_{0}-\Delta m_{1}}{\Delta m_{0}}\times 100\%$$

Herein, ρ is the density of N80 steel (7.85 g/cm^3^), t is the corrosion time (h), $\Delta m_{0}$ stands for the mass loss of the coupon in the blank test (without inhibitor, g), and $\Delta m_{1}$ refers to the mass loss of the coupon in the test with the inhibitor (g). All static weight loss experiments were conducted in triplicate to guarantee reproducibility. Specifically, comparative weight loss data for N80 steel protected with TN-IM or STD-MC at different concentrations (Table S1), CO_2_ pressures (Table S2), corrosion temperatures (Table S3), corrosion times (Table S4), and NaCl concentrations (Table S5) were recorded.

### 2.5. Electrochemical Measurements

PDP and EIS were used to measure corrosion electrochemical reactions using a standard three-electrode system. The working electrode was the N80 steel electrode (exposed area 1 cm^2^), the auxiliary electrode was the platinum sheet, and the saturated reference electrode was the saturated calomel electrode (SCE). The working electrode was prepared by embedding the N80 steel coupon in epoxy resin (A:B = 3:1, mass ratio), grinding with 2000-mesh sandpaper, degreasing with acetone, and rinsing with deionized water before use. The test medium was a 3.5 wt% NaCl solution saturated with CO_2_, with different concentrations of TN-IM (0.03, 0.06, 0.09, 0.12, and 0.15 mmol/L) as the inhibitor. All measurements were carried out at 30 °C. First, the open-circuit potential (OCP) of the working electrode was measured for 30 min until it reached a steady state (potential variation < 5 mV/min). Then, EIS measurements were performed in the frequency range of 100 kHz to 0.1 Hz with an alternating current (AC) amplitude of 5 mV. Potentiodynamic polarization curves were measured immediately after EIS tests at a scan rate of 0.01 mV/s over a potential range of −200 to + 200 mV vs. OCP.

The corrosion inhibition efficiency from potentiodynamic polarization ($\eta_{2}$, %) and EIS ($\eta_{3}$, %) were calculated using Equation (3) and Equation (4) [27], respectively.

(3) $$\eta_{2}=\frac{I_{corr,0}-I_{corr,\;1}}{I_{corr,\;0}}\times 100\%$$

(4) $$\eta_{3}=\frac{R_{p,1}-R_{p,\;0}}{R_{p,\;1}}\times 100\%$$

where $I_{corr,0}$ and $I_{corr,\;1}$ are the corrosion current densities (μA/cm^2^) of N80 steel in the blank solution and inhibitor-containing solution, respectively (obtained by Tafel extrapolation), and $R_{p,0}$ and $R_{p,1}$ are the polarization resistances (Ω·cm^2^) of N80 steel in the solutions without and with corrosion inhibitors, respectively (obtained by EIS fitting). Equivalent circuit fitting of the EIS spectra was carried out using Zview2 software with the appropriate circuit models.

### 2.6. Methods for Surface Morphology and Composition Analysis

The surface morphology and elemental composition of N80 steel coupons after corrosion were characterized by SEM (VEGA 3SBU, NETZSCH, Waldkraiburg, Germany) at an accelerating voltage of 20 kV and EDS (attached to the SEM, analysis range 0~20 keV), respectively. The test conditions for surface characterization were consistent with the optimal conditions from the static weight loss tests: 3.5 wt% NaCl solution, 0.6 MPa CO_2_, 60 °C, 24 h, and 0.15 mmol/L inhibitor (TN-IM or STD-MC). The blank test (without inhibitor) was also performed under the same conditions for comparison. All samples were dried for 2 h in a vacuum oven at 40 °C prior to SEM/EDS characterization to avoid surface oxidation.

X-ray photoelectron spectroscopy (XPS, Thermo Fisher Scientific, Waltham, MA, USA) was employed to analyze the surface composition of N80 specimens after blank corrosion and after TN-IM addition. The binding energy was calibrated using the C1s peak (284.6 eV) [28]. The acquired XPS data was fitted using Avantage software (6.8.0 Build 00054, Thermo Fisher Scientific, Waltham, USA).

### 2.7. Quantum Chemical Calculations

This study employs quantum chemical (or density functional theory, DFT) calculations to systematically investigate the adsorption behavior and intrinsic characteristics of TN-IM on metal surfaces. Geometric optimization of the inhibitor molecules was carried out using the 6–31G (d,p) basis set. Gaussian 9 calculations were performed on the optimized TN-IM structure (Figure S2) to determine the highest occupied molecular orbital (HOMO), lowest unoccupied molecular orbital (LUMO), and corresponding energy gap (ΔE) [29,30] (Figure S3), as well as to visualize the electrostatic potential distribution of TN-IM (Figure S4). Significantly, it should be noted that, in corrosive environments with high ionic strength, the descriptors obtained from DFT calculations should be regarded as supplementary qualitative indicators rather than direct quantitative prediction tools.

### 2.8. Adsorption Kinetic Calculations

Typically, adsorption isotherms can be used to evaluate the adsorption capacity of corrosion inhibitors on metal surfaces. The adsorption of dissolved corrosion inhibitors on metal surfaces follows adsorption isotherm equations, as shown in Equation (5). The adsorption of TN-IM on the surface of N80 test specimens is monolayer adsorption, where the surface coverage is approximately equal to the corrosion inhibition rate obtained from corrosion weight loss tests [31].

(5) $$\frac{c}{\theta }=c+\frac{1}{K_{\mathrm{ads}}}$$

Herein, c is the mass concentration of TN-IM (g/L), *θ* is the coverage on the specimen surface and *K*_ads_ is the Langmuir adsorption equilibrium constant. The corrosion inhibition effect of TN-IM is primarily achieved through its characteristic adsorption process on the N80 specimen surface. To investigate its adsorption behavior, adsorption isotherms at a specific temperature were fitted as shown in Figure S5. The slope was approximately equal to 1, indicating that TN-IM adsorption on the N80 specimen surface conforms to the Langmuir adsorption isotherm. *K*_ads_ is related to the standard Gibbs free energy of adsorption, as expressed by Equation (6) [32]:

(6) $$K_{ads}=\frac{1}{55.5}exp(\frac{-\Delta G_{ads}^{\theta }}{RT})$$

where 55.5 mol/L is the solvent water concentration; *R* is the gas constant with the unit of J/(mol/K); and *T* is the temperature (K). The standard Gibbs free energy of adsorption can be calculated using the above equation as given in Table S6. If *ΔG*^θ^ < 0, it indicates spontaneous adsorption of the corrosion inhibitor on the N80 steel surface. If *ΔG*^θ^ > −20 kJ/mol, it indicates physical adsorption of the inhibitor on the steel surface. If *ΔG*^θ^ < −40 kJ/mol, it indicates chemical adsorption of the inhibitor on the steel surface.

Furthermore, the thermodynamic adsorption parameters of TN-IM on the surface of N80 specimens may be calculated using Equation (7) [32]:

(7) $$lnK_{ads}=ln\frac{1}{55.5}-\frac{{∆H}_{ads}^{0}}{RT}+\frac{{∆S}_{ads}^{0}}{R}$$

wherein, ${∆H}_{ads}^{0}$ is the enthalpy of adsorption, kJ/mol, and ${∆S}_{\mathrm{ads}}^{0}$ is the entropy of adsorption, J/(K·mol).

## 3. Results and Discussion

### 3.1. Structural Characterization of TN-IM

#### 3.1.1. FT-IR of TN-IM and STD-MC

**Figure 2:**
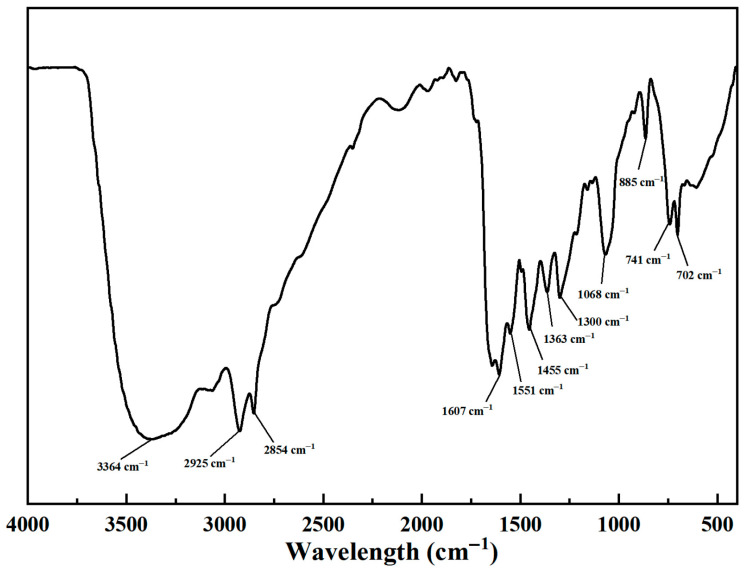
FT-IR spectrum of TN-IM.

The FT-IR spectrum of TN-IM is depicted in Figure 2. The broad peak at 3364 cm^−1^ is attributed to the stretching vibration of N-H bonds in the imino group of the imidazoline ring [33]. The two peaks at 2925 cm^−1^ and 2854 cm^−1^ correspond to the asymmetric and symmetric stretching vibrations of –CH_2_– groups in the alkyl chain and benzyl group [34], respectively. The sharp peak at 1607 cm^−1^ is characteristic of –C=N– stretching vibration in the imidazoline ring [35], which confirms the successful formation of the imidazoline ring structure in TN-IM. The adsorption peaks at 1551 cm^−1^ and 1455 cm^−1^ are assigned to the stretching vibrations of –C=C– bonds in the benzene ring of the benzyl group [36]. The peak at 1363 cm^−1^ is associated with the in-plane bending vibration of –CH_2_– groups [36]. The peak at 1300 cm^−1^ corresponds to the stretching vibration of–C–N– bonds in the quaternary ammonium group, which indicates that the successful quaternization reaction processed successfully [37]. The peak at 1068 cm^−1^ is attributed to the in-plane bending vibration of the –OH groups in the hydroxyethyl side chain [38,39]. The peaks at 885 cm^−1^ and 741 cm^−1^ are assigned to the out-of-plane bending vibrations of C–H bonds in the alkyl chain [33,40]. The peak at 702 cm^−1^ is the characteristic absorption peak of the benzyl group in the corrosion inhibitor [33], further confirming the successful introduction of the benzyl group through quaternization reaction. All the observed characteristic absorption peaks are consistent with the designed molecular structure of TN-IM, which demonstrates the successful synthesis of the target dual-imidazoline ring quaternary ammonium salt. The core functional groups of STD-MC, including the imidazoline ring, quaternary ammonium moiety, and long alkyl chain, are identical to those of TN-IM, with the only distinction lying in the number of imidazoline rings. Consequently, the FTIR spectrum of STD-MC is given in Figure S1.

#### 3.1.2. H-NMR of TN-IM

**Figure 3:**
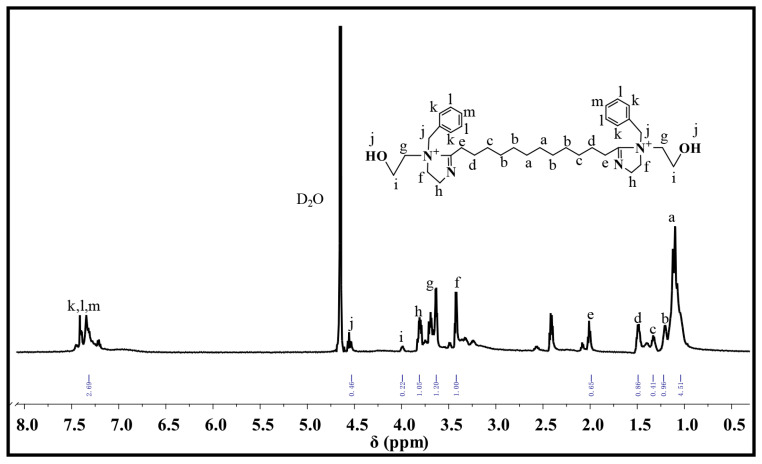
^1^HNMR spectrum of TN-IM.

The ^1^H-NMR spectrum of TN-IM (D_2_O, 200 MHz) is shown in Figure 3, and the chemical shifts (δ) are assigned as follows: δ 1.16–1.33 ppm (peaks a, b, c) correspond to the proton signals of the methylene groups (–CH_2_–) in the tetradecanedioic acid-derived alkyl chain; δ 1.50–1.54 ppm (peak d) is the proton signal of the methylene groups adjacent to the imidazoline ring; δ 2.01–2.11 ppm (peak e) is the proton signal of the methylene groups in the hydroxyethyl side chain [41,42,43]; δ 3.30–3.42 ppm (peak f) and 3.81–3.95 ppm (peak h) are the proton signals of the methylene groups linked to the quaternary nitrogen atom (–(N^+^)CH_2_CH_2_NC–) in the imidazoline ring [33,38]; δ 3.43–3.63 ppm (peak g) and 3.97–3.99 ppm (peak i) are the proton signals of the hydroxyethyl methylene groups (–(N^+^)CH_2_CH_2_OH) [44]; δ 4.50–4.53 ppm (peak j) is the proton signal of the methylene group linking the quaternary nitrogen atom and the benzene ring (–(N^+^)CH_2_C_6_H_5_) [42]; δ 7.16–7.40 ppm (peaks k, l, m) are the proton signals of the aromatic hydrogen atoms in the benzene ring [45].

The integral area of each proton peak was calculated, and the results showed that the integral area ratio of the proton peaks on the imidazoline ring (peaks f, h) was 1.00:1.05, which is consistent with the hydrogen atom ratio in the dual-imidazoline ring structure. The total integral area of all proton peaks was 14.01, corresponding to the total number of hydrogen atoms (56) in the TN-IM molecule with a 1:4 ratio, which further confirms the successful synthesis of the dual-imidazoline ring quaternary ammonium salt with the designed molecular structure.

### 3.2. Corrosion Inhibition Performance of TN-IM and STD-MC

#### 3.2.1. Concentration Effects of Inhibitors

**Figure 4:**
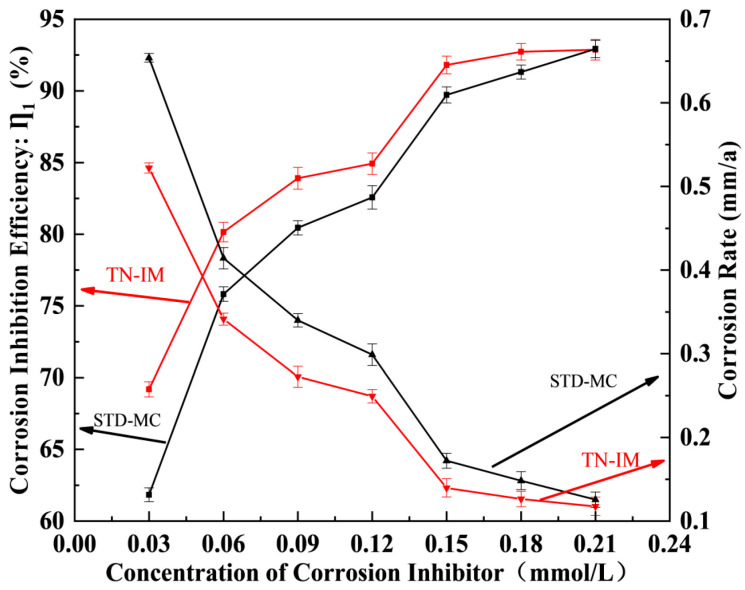
Concentration effects of TN-IM and STD-MC on their corrosion inhibition properties.

The effect of inhibitor concentration (0.03~0.21 mmol/L) on the inhibitory effect of TN-IM and STD-MC for N80 steel in the 3.5 wt% NaCl solution under 0.6 MPa CO_2_ at 60 °C for 24 h is shown in Figure 4. Both TN-IM and STD-MC exhibit a decrease in the corrosion rate and an increase in corrosion inhibition efficiency with rising inhibitor concentration, which is attributed to the increased coverage of inhibitor molecules on the metal surface with the increase in concentration. At a concentration of 0.15 mmol/L, TN-IM achieves a corrosion inhibition efficiency of 91.76% with a corrosion rate of 0.1395 mm/a, which is 1.86% higher in IE and 0.0329 mm/a lower regarding the corrosion rate than STD-MC (IE = 89.90%, $r_{c}$ = 0.1724 mm/a). For 0.03 mmol/L of TN-IM, the corrosion rate is relatively high (0.5226 mm/a), because the adsorption layer of TN-IM covering the N80 coupons is incomplete at this concentration, leading to severe surface corrosion on N80 coupons exposed to the corrosive solution [46]. When the concentration exceeds 0.15 mmol/L, the increase in IE becomes slow, indicating that the surface coverage of TN-IM on N80 approaches saturation [4]. TN-IM achieves an IE of 93.06% at 0.21 mmol/L with a corrosion rate of 0.1178 mm/a, and further increasing the concentration has no significant effect on the corrosion inhibition performance. This saturation behavior is a typical characteristic of organic corrosion inhibitors adsorbing onto metallic surfaces, where the active sites on the metal surface are fully covered by inhibitor molecules at the critical micelle concentration.

#### 3.2.2. Effects of CO_2_ Pressure

**Figure 5:**
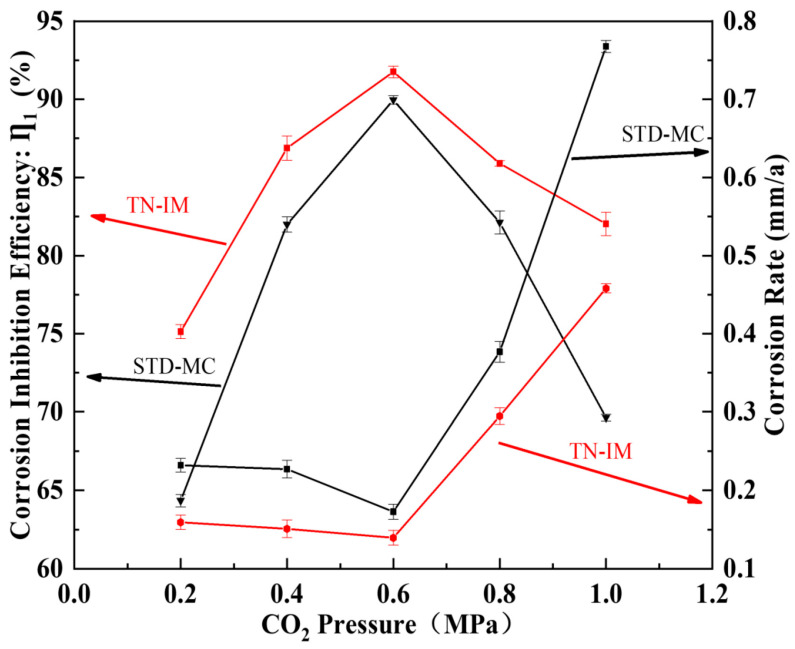
Effects of CO_2_ pressure variations on corrosion inhibition performance of TN-IM and STD-MC.

The effects of CO_2_ pressure (0.2~1.0 MPa) on the corrosion inhibition performance of TN-IM and STD-MC (0.15 mmol/L) are shown in Figure 5, and the comparative weight loss of N80 with and without 0.15 mmol/L of TN-IM or STD-MC at different CO_2_ pressures is given in Table S7. The experiment was conducted in the 3.5 wt% NaCl solution at 60°C for a duration of 24 h. The IE of both inhibitors first increases and then decreases with the increase in CO_2_ pressure, and TN-IM exhibits better performance than STD-MC at all tested pressures. At a CO_2_ pressure of 0.6 MPa, the IE of TN-IM reaches the maximum value of 91.76%, while at 0.4 MPa, TN-IM has an IE of 86.82% (corrosion rate $r_{c,TN-IM}$ = 0.1508 mm/a), which is 4.83% higher than that of STD-MC (IE = 81.99%, $r_{c,STM-MC}$ = 0.2270 mm/a) and the condition added without TN-IM or STD-MC ($r_{c,N80}$ = 1.6624 mm/a).

The initial increase in IE with CO_2_ pressure is due to the formation of carbonic acid (H_2_CO_3_) when CO_2_ dissolves in the aqueous solution, which decreases the pH of the solution and increases the electronegativity of the N80 steel surface. This enhances the electrostatic adsorption of the positively charged quaternary ammonium cations of TN-IM on the metal surface, and the in situ formed FeCO_3_ film synergistically improves the corrosion protection effect with the inhibitor adsorption film [47]. However, when the CO_2_ pressure exceeds 0.6 MPa, the concentration of H_2_CO_3_ increases significantly, leading to a sharp decrease in the solution pH and a high H^+^ concentration. The H^+^ ions dissolve the protective FeCO_3_ film, and the dissolution rate exceeds the formation rate, resulting in the destruction of the FeCO_3_ film [48]. In addition, the high concentration of HCO_3_^−^/CO_3_^2−^ ions compete with TN-IM molecules for the active adsorption sites on the metal surface, leading to a decrease in the coverage of the inhibitor adsorption film and a reduction in IE [49].

#### 3.2.3. Effects of Temperature

**Figure 6:**
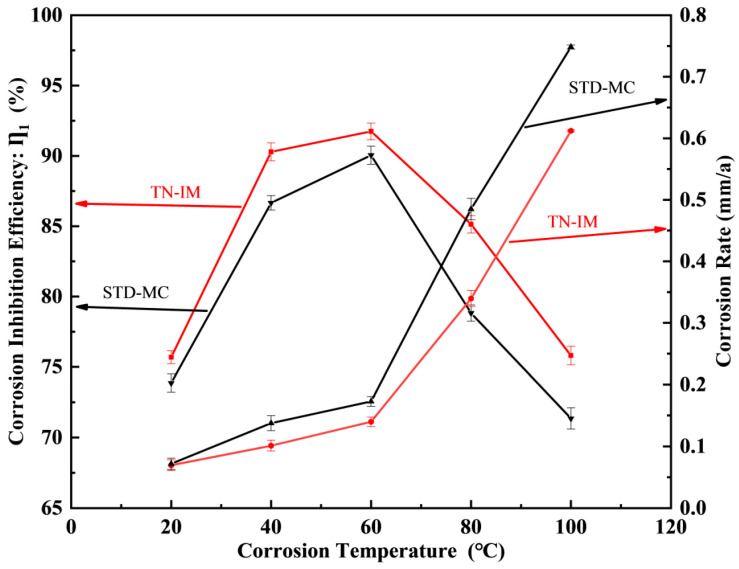
The effects of temperature variations on the corrosion inhibition performance of TN-IM and STD-MC.

The effects of temperature (20~100 °C) on the corrosion inhibition performance of TN-IM and STD-MC (0.15 mmol/L) are shown in Figure 6. Also, the comparative weight loss of N80 with and without 0.15 mmol/L of TN-IM or STD-MC at different temperatures are given in Table S8. The experiment was conducted in the 3.5 wt% NaCl solution under a CO_2_ pressure of 0.6 MPa for a duration of 24 h. The IE of both inhibitors first increases and then decreases with the increase in temperature, while the corrosion rate shows an overall increasing trend. TN-IM maintains a higher IE than STD-MC at all tested temperatures, with the maximum IE at 60 °C. At 40 °C, TN-IM has an IE of 90.14% (${r\prime }_{c,TN-IM}$ = 0.1101 mm/a), which is 3.45% higher than that of STD-MC (IE = 86.69%, ${r\prime }_{c,STM-MC}$ = 0.1471 mm/a) and the condition added without TN-IM or STD-MC (${r\prime }_{c,N80}$ = 1.0236 mm/a). The initial increase in IE with temperature is due to the accelerated migration rate of inhibitor molecules from the bulk solution to the metal surface at moderate temperatures, which promotes the adsorption of TN-IM molecules on the active sites and the formation of a dense adsorption film [50]. However, when the temperature exceeds 60 °C, the thermal motion of the adsorbed TN-IM molecules is intensified, leading to inhibitor desorption off the metal surface and the destruction of the adsorption film. In addition, high temperatures may cause the decomposition, oxidation, or hydrolysis of partial TN-IM molecules, resulting in the loss of active adsorption groups and a significant reduction in IE. The overall increase in the corrosion rate with temperature is due to the accelerated electrochemical corrosion reaction of N80 steel in the CO_2_-containing corrosive medium at high temperatures [51].

#### 3.2.4. Effects of Corrosion Time

**Figure 7:**
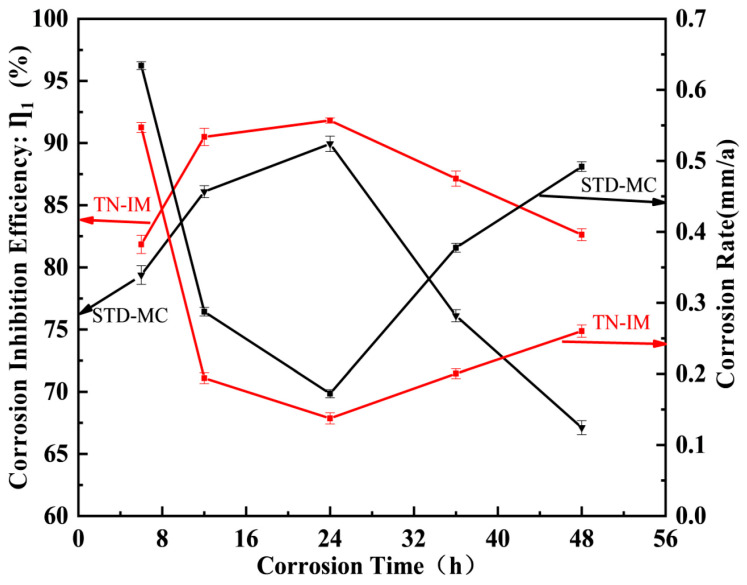
The effects of corrosion time on the corrosion inhibition performance of TN-IM and STD-MC.

The effects of corrosion time (0~48 h) on the corrosion inhibition performance of TN-IM and STD-MC (0.15 mmol/L) are shown in Figure 7. The experiment was conducted in the 3.5 wt% NaCl solution at 60°C and a CO_2_ pressure of 0.6 MPa. The IE of both inhibitors first increases and then decreases as the corrosion time progresses, while the corrosion rate first decreases and then increases. TN-IM exhibits superior performance to STD-MC at all tested times, with the maximum IE at 24 h (91.76%). At 36 h, TN-IM has an IE of 87.25% (${r\prime \prime }_{c,TN-IM}$ = 0.2007 mm/a), which is 11.06% higher than that of STD-MC (IE = 76.19%, ${r\prime \prime }_{c,STM-MC}$ = 0.3778 mm/a), and the condition added without TN-IM or STD-MC (${r\prime \prime }_{c,N80}$ = 1.6007 mm/a) can be estimated from Table S4.

The initial increase in IE with corrosion time is because the adsorption of TN-IM molecules on the N80 steel surface is a time-dependent process, and the inhibitor gradually occupies the surface-active sites to form a dense and stable adsorption film within 24 h [52]. When the corrosion time exceeds 24 h, the adsorbed TN-IM molecules gradually desorb due to thermal motion and the scouring of the corrosive medium, leading to the deterioration of the adsorption film. In addition, the long-term corrosion reaction produces a large amount of corrosion products, which accumulate on the metal surface and block the adsorption of fresh inhibitor molecules, resulting in a decrease in IE and an increase in the corrosion rate [53].

#### 3.2.5. Concentration Effects of NaCl Solution

**Figure 8:**
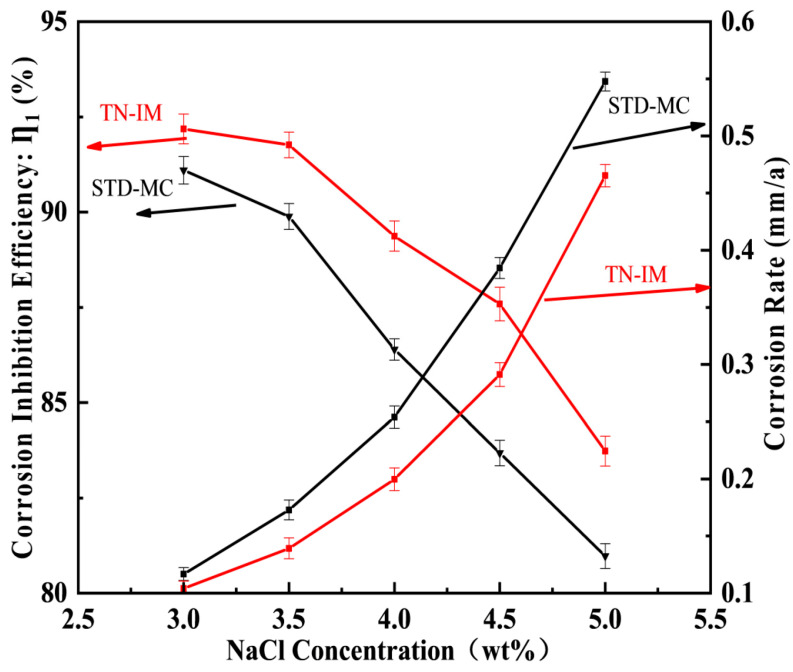
The effects of NaCl concentration on the corrosion inhibition performance of TN-IM and STD-MC (0.15 mmol/L).

The effects of NaCl concentration (3.0~5.5 wt%) on the corrosion inhibition performance of TN-IM and STD-MC (0.15 mmol/L) are shown in Figure 8. The experiment was conducted at 60 °C and a CO_2_ pressure of 0.6 MPa for 24 h. The IE of both inhibitors decreases gradually with the increase in NaCl concentration, while the corrosion rate increases continuously, and TN-IM still shows better performance than STD-MC at all tested concentrations. At 4.0 wt% NaCl, TN-IM has an IE of 89.34% (${r\prime \prime \prime }_{c,TN-IM}$ = 0.1997 mm/a), which is 2.93% higher than that of STD-MC (IE = 86.41%, ${r\prime \prime \prime }_{c,STM-MC}$ = 0.2534 mm/a), and the condition added without TN-IM or STD-MC (${r\prime \prime \prime }_{c,N80}$ = 1.8834 mm/a) can be estimated from Table S5.

The decrease in IE with the increase in NaCl concentration is mainly ascribed to the effect of Cl^−^ ions. On the one hand, the N80 steel surface is positively charged in the aqueous solution, and the Cl^−^ ions with strong electronegativity are first adsorbed on the metal surface via electrostatic attraction to form a pre-adsorption layer, which hinders the adsorption of positively charged TN-IM molecules on the metal surface [54]. On the other hand, Cl^−^ ions can penetrate the inhibitor adsorption film and destroy the chemical bonds between the inhibitor molecules and the metal surface, leading to the desorption of inhibitor molecules and the occurrence of localized corrosion (e.g., pitting corrosion) [55]. In addition, the high concentration of Cl^−^ ions accelerates the electrochemical corrosion reaction of N80 steel by enhancing the medium’s ability to conduct ions and facilitating electron transfer across the interface.

### 3.3. Electrochemical Corrosion Inhibition Mechanism

#### 3.3.1. Potentiodynamic Polarization Curves

**Figure 9:**
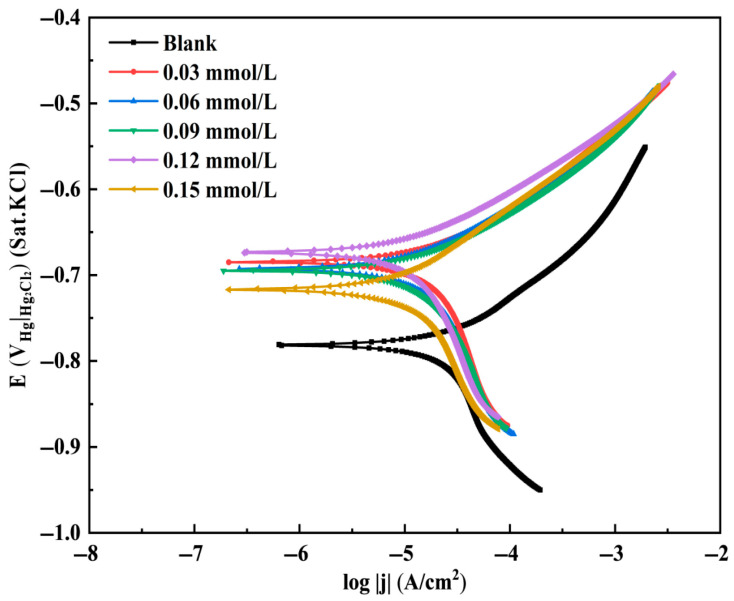
The potentiodynamic polarization curves of N80 steel in 3.5 wt% NaCl solution saturated with CO_2_ with different concentrations of TN-IM (0, 0.03, 0.06, 0.09, 0.12, 0.15 mg/L).

The potentiodynamic polarization curves for N80 steel in the CO_2_-saturated 3.5 wt% NaCl solution with various TN-IM concentrations (0.03, 0.06, 0.09, 0.12, and 0.15 mmol/L) are presented in Figure 9. Table 2 lists the corresponding electrochemical parameters (corrosion potential *E*_corr_, anodic Tafel slope $\beta_{a}$, cathodic Tafel slope $\beta_{c}$, corrosion current density *I*_corr_, and inhibition efficiency $\eta_{2}$) obtained via Tafel extrapolation.

It can be seen that the addition of TN-IM leads to a significant decrease in both anodic and cathodic current densities, without changing the shape of the polarization curves, indicating that TN-IM inhibits both the anodic dissolution of Fe and the cathodic reduction of H_2_CO_3_/H^+^ and does not change the electrochemical corrosion mechanism of N80 steel in the CO_2_-containing corrosive medium [56]. The *E*_corr_ of N80 steel shifts positively with the addition of TN-IM, and the shift value is less than 85 mV, indicating that TN-IM acts as a mixed-type corrosion inhibitor. The positive shift of *E*_corr_ also indicates that TN-IM has a predominant inhibitory effect on the anodic reaction, which is attributed to the adsorption of TN-IM molecules on the anodic active sites of N80 steel, inhibiting the dissolution of Fe into Fe^2+^ and the formation of corrosion products [57,58].

The *I*_corr_ of N80 steel decreases significantly with the increase in TN-IM concentration, from 41.02 μA/cm^2^ (blank) to 5.12 μA/cm^2^ (0.15 mmol/L TN-IM), and the corresponding $\eta_{2}$ increases from 0 to 87.52%. This is consistent with the results of the static weight loss method, confirming that TN-IM effectively inhibits the electrochemical corrosion of N80 steel by forming an adsorbed layer on the metal.

#### 3.3.2. Electrochemical Impedance

**Figure 10:**
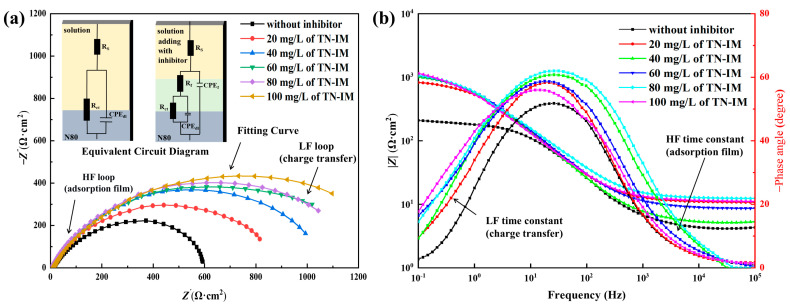
Nyquist plots with equivalent circuit (**a**) and Bode plots (**b**) for TN-IM.

**Figure 11:**
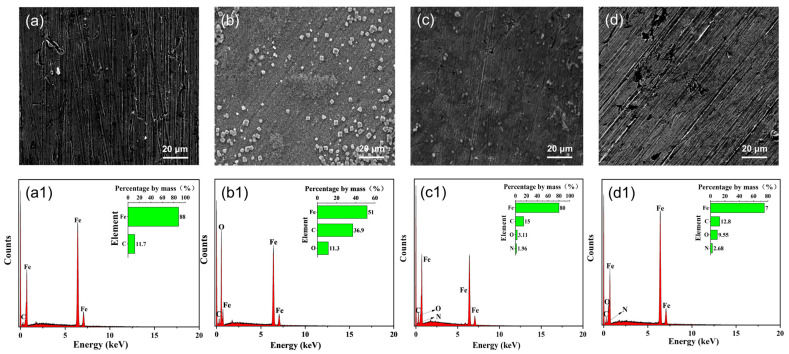
Surface and EDS spectrum of N80 under different conditions ((**a,a1**) before corrosion, (**b**,**b1**) after corrosion without inhibitor, (**c**,**c1**) after corrosion adding with STD-MC, (**d**,**d1**) after corrosion adding with TN-IM).

The discrete symbols represent the experimental EIS data, whereas the corresponding fitting curves obtained from Zview2 software were initially plotted with the same color as the experimental data points, as given in Figure 10. The Nyquist plots in Figure 10a reveal a single capacitive loop for the blank solution and two distinct loops in the presence of TN-IM. The diameter of these loops increases progressively with TN-IM concentration, indicating improved corrosion resistance arising from the adsorbed TN-IM layer on the specimen surface [59]. The Bode plots (Figure 10b) show that the impedance modulus |Z| at a low frequency increase with the increase in TN-IM concentration, and the phase angle peak becomes broader and shifts to lower frequencies, indicating the formation of a stable inhibitor adsorption film on the metal surface. The appearance of two-time constants in the EIS spectra of the inhibitor-containing solutions confirms the formation of the adsorption film with its own electrochemical characteristics, which is consistent with the equivalent circuit model (inserted in Figure 11a) [60]. Here, *R*_s_ denotes the resistance of the corrosive medium, *R*_ct_ represents the charge transfer resistance on the surface of N80, and *R*_f_ signifies the resistance associated with the formation of the corrosion inhibitor adsorption film on the specimen surface. CPE_dl_ and CPE_f_ respectively denote the constant phase angle components of the double layer and adsorption film [61]. The $R_{ct}$ value increases significantly from 157.9 Ω cm^2^ (blank) to 1246.0 Ω cm^2^ (0.15 mmol/L TN-IM), and the $R_{f}$ value also increases with the increase in TN-IM concentration, indicating that TN-IM molecules form a dense adsorption film on the N80 steel surface, which significantly inhibits the charge transfer process of the electrochemical corrosion reaction [62]. CPE_dl_ decreases with the increase in TN-IM concentration, which is due to TN-IM molecules displacing water molecules from the electrical double layer [63], leading to a decrease in the capacitance of the electrical double layer and the thickening of the double layer, further confirming the adsorption of TN-IM on the metal surface. The $\eta_{3}$ value increases from 0 to 87.07% with the increase in TN-IM concentration, which is consistent with the results of potentiodynamic polarization and static weight loss methods, indicating the reliability of the experimental results. The EIS results confirm that the corrosion inhibition mechanism of TN-IM is mainly based on inhibitor molecules adsorbing onto the N80 steel’s active sites, forming a physical and chemical adsorption film that inhibits charge transfer during the electrochemical corrosion reaction, thus retarding the corrosion of N80 steel in the CO_2_-containing corrosive medium.

### 3.4. Surface Morphology and Composition Analysis

#### 3.4.1. SEM Analysis

The SEM micrographs of N80 steel coupons under different conditions (polished, blank corrosion, TN-IM, and STD-MC) are shown in Figure 11. The polished N80 surface is smooth and flat with clear grinding marks, without any corrosion products or defects. The blank corrosion sample shows a severely corroded surface, which is covered with a large amount of irregular corrosion products (square and needle-shaped), and the surface is rough with deep corrosion grooves, indicating that severe electrochemical corrosion occurs on N80 steel in the CO_2_-containing 3.5 wt% NaCl solution without an inhibitor.

For the sample with TN-IM added, the surface is relatively smooth with only a small amount of fine corrosion products and shallow corrosion grooves, and the original grinding marks are still clearly visible, indicating that TN-IM effectively inhibits the corrosion of N80 steel and forms a protective adsorption film on the metal surface. For the sample with STD-MC added, the surface has more obvious corrosion grooves and a larger amount of corrosion products compared with the TN-IM sample, although the corrosion degree is lower than that of the blank sample [64]. This further confirms that TN-IM has a better corrosion inhibition performance than STD-MC, which agrees with the electrochemical measurements and static weight loss.

#### 3.4.2. EDS Analysis

The EDS spectra and elemental composition of N80 steel coupons under different conditions are shown in Figure 11 and Table 3. The polished N80 steel surface only contains C and Fe elements (the intrinsic elements of the steel), with no other elements detected, which is attributed to that the EDS results are only semi-quantitative and indicative rather than fully accurate. The blank corrosion sample contains C, Fe, and O elements, with the Fe content significantly decreased and the O content increased, which is due to the formation of iron oxide/hydroxide and FeCO_3_ corrosion products on the steel surface during the CO_2_ corrosion reaction [65].

Both the TN-IM and STD-MC samples contain C, Fe, O, and N elements, and the N content in the TN-IM sample (5.99 mol%) is higher than that in the STD-MC sample (4.63 mol%). The presence of the N element confirms that both inhibitor molecules adsorb on the N80 steel surface, and the higher N content in the TN-IM sample indicates a higher adsorption amount of TN-IM molecules on the metal surface. In addition, the C/Fe molar ratio decreases from the STD-MC sample to the TN-IM sample, demonstrating that the TN-IM adsorption film effectively hinders corrosion product buildup on the metal surface. The EDS results confirm that TN-IM has a stronger adsorption capacity on the N80 steel surface than STD-MC, which is the main reason for its better corrosion inhibition performance [66].

#### 3.4.3. XPS Analysis

The XPS spectra of N80 specimens after 24 h of corrosion in a 3.5 wt% NaCl solution, with and without the addition of 0.15 mmol/L TN-IM in saturated CO_2_, are presented in Figure S6. For the Fe2p spectrum in Figure S6a1,b1, seven peaks appear at 707.2 eV, 711.0 eV, 714.5 eV, 719.2 eV, 724.2 eV, 727.4 eV and 732.3 eV, attributed respectively to Fe, FeO/Fe_2_O_3_, Fe^2+^, Fe^3+^, Fe_3_O_4_, Fe^2+,^ and Fe^3+^ [67]. The appearance of the Fe_2_O_3_, FeO, Fe_3_O_4_, and FeOOH peaks may be related to the oxidation of Fe(II) in air. In the C1s spectrum, as shown in Figure S6a2,b2, a narrow main peak at 284.6 eV can be attributed to C=C, the peak at 286.3 eV to C=O, and the peak at 288.1 eV to C=O/FeCO_3_ [50]. For the O1s spectrum in Figure S6a3,b3, TN-IM exhibits an additional peak at 534.7 eV compared to the blank phase; this is because the peak corresponds to the C-O peak on the TN-IM corrosion inhibitor. Furthermore, in the N1s spectrum (Figure S6c), a peak at 400.1 eV appears on the sample surface in the solution containing TN-IM; this can be attributed to C-N on the TN-IM corrosion inhibitor and N-Fe on the surface of the TN-IM and N80 test specimens [68].

### 3.5. Inhibition Mechanism of TN-IM

Figure S2 depicts the optimized geometric structure and frontier molecular orbital distribution of TN-IM. It is evident that the HOMO orbitals of TN-IM are predominantly distributed across the benzene ring, whilst the LUMO orbitals are primarily distributed across the imidazoline ring within the TN-IM molecule. Figure S3 presents the orbital energy levels of TN-IM: *E*_HOMO_ = −10.850 eV, *E*_LUMO_ = −5.153 eV, and Δ*E* = 5.697 eV. These data indicate that TN-IM can undergo greater electron transfer with carbon steel surfaces, interacting with the vacant d orbitals of iron to form feedback bonds. This enhances the stability of TN-IM adsorption on metallic surfaces [69].

According to Koopman’s theorem, the ionization potential (*I*) and electron affinity (*A*) associated with the *E*_HOMO_ and *E*_LUMO_ of the inhibitor molecule are defined as shown in Equations (8) and (9) [70,71]:

*I =* −*E*_HOMO_ (8)

*A =* −*E*_LUMO_ (9)

The absolute electronegativity (*X*), overall hardness (*γ*), and fraction of electrons transferred from the inhibitor to the metal surface (*ΔN*) are given by Equation (10), Equation (11), and Equation (12), respectively [65,66,71]:

(10) $$X=\frac{I+A}{2}$$

(11) $$\gamma =\frac{I-A}{2}$$

(12) $$∆N=\frac{X_{Fe}-X}{2\left(\gamma_{Fe}+\gamma \right)}$$

where *X*_Fe_ = 7 eV/mol and *γ*_Fe_ = 0 eV/mol.

Stable molecular structures exhibit larger ΔE values; thus, electron systems with greater hardness are more stable than those with lower hardness. A higher *X* value indicates a greater tendency for the molecule to accept electrons and a reduced capacity to donate them. Should *ΔN* be less than 3.6, this signifies that as the electron-donating capacity of the metal surface increases, the corrosion inhibition efficiency of the inhibitor also rises [72]. In this study, TN-IM exhibits a *ΔN* value of −0.1758, indicating its capacity to donate electrons to the metal surface, thereby facilitating the formation of an adsorbed protective film. Compared to reported monoimidazoline quaternary ammonium salt corrosion inhibitors [67], TN-IM exhibits a lower *E_LUMO_* and a higher *X* value. This indicates that TN-IM readily accepts electrons from the metal surface, demonstrating stronger electron-accepting capacity. This enhances the formation of covalent bonds and adsorption-protective films, thereby increasing corrosion inhibition efficiency.

Electrostatic potential (ESP) analysis may be employed to investigate the local polarity of TN-IM molecules and the electrostatic interactions between the molecules and the external environment. Figure S4 depicts the electrostatic potential distribution of TN-IM. Within the figure, the majority of the TN-IM spectrum is covered in blue, indicating a tendency to accept electrons from the three-dimensional orbitals of iron atom vacancies on the steel surface, thereby favoring the adsorption of the protective film [73,74]. Simultaneously, the entirely positive electrostatic potential regions of TN-IM confirm its status as an electrophilic inhibitor. This mechanism involves chemical adsorption dominated by covalent bonds through charge sharing or transfer, consistent with adsorption kinetic calculations.

Furthermore, the dipole moment (μ) is widely employed to characterize the polarity of inhibitor molecules. This parameter correlates with hydrophobicity, where lower values indicate stronger hydrophobic properties. Calculations yield a μ value of 5.4070 D for TN-IM. Compared to the μ value of water molecules (1.88 D), TN-IM is more likely to displace water molecules adsorbed on the N80 test specimen surface [75]. This indicates that TN-IM exhibits excellent corrosion inhibition performance by adsorbing onto the N80 test specimen surface and impeding the diffusion of corrosive media. It should be noted that, in corrosive environments with high ionic strength, the descriptors obtained from DFT calculations should be regarded as supplementary qualitative indicators rather than direct quantitative prediction tools.

Moreover, the corrosion rates and inhibition efficiencies of N80 specimens in different concentrations of TN-IM were measured by varying the corrosion temperature. The Langmuir adsorption equilibrium constant *K* under these conditions was calculated using Equation (5), with the results presented in Figure S5a–e.

By continuing with Equations (6) and (7), the adsorption standard Gibbs free energy ΔG^θ^, adsorption process enthalpy ${∆H}_{ads}^{0}$ and adsorption process entropy ${∆S}_{ads}^{0}$ can be calculated through fitting. The calculated results are presented in Table S6.

Table S6 indicates that the adsorption enthalpy ${∆H}_{ads}^{0}$ of TN-IM on the N80 specimen surface is 9.0024 kJ/mol, demonstrating that TN-IM adsorption onto the N80 surface is an endothermic process. Elevating temperature facilitates the adsorption of the corrosion inhibitor onto the N80 specimen surface, which also explains the results from the corrosion weight loss experiment regarding the influence of different temperatures on the corrosion inhibition efficacy of TN-IM. The entropy change for adsorption, ${∆S}_{ads}^{0}$, is 156.4344 J/(K·mol), which is considered the driving force for TN-IM adsorption onto the N80 specimen surface. Calculations of ${∆G}_{ads}^{\theta }$ for TN-IM at different temperatures all exceed 40 kJ/mol, indicating that TN-IM primarily undergoes chemisorption via charge sharing to form covalent bonds.

## 4. Conclusions

A novel dual-imidazoline ring quaternary ammonium salt corrosion inhibitor (TN-IM) was successfully synthesized via sequential amidation (160 °C, 4 h), cyclization (220 °C, 3 h), and quaternization (70 °C, 3 h) reactions, using hydroxyethyl ethylenediamine, tetradecanedioic acid, and benzyl chloride as feedstocks. The chemical structure of TN-IM was confirmed by FTIR and ^1^H NMR. Notably, the integral area ratio of the proton peaks corresponding to the imidazoline ring in the ^1^H NMR spectrum was consistent with the hydrogen atom ratio in the dual-imidazoline ring structure, further verifying the successful formation of the target dual-imidazoline ring structure. Static weight loss tests were conducted to evaluate the corrosion inhibition performance for N80 carbon steel in a 3.5 wt% NaCl solution saturated with 0.6 MPa CO_2_ at 60 °C for 24 h. The results showed that TN-IM exhibits exceptional corrosion inhibition performance, achieving a maximum inhibition efficiency of 91.76% and a corrosion rate of 0.1395 mm/a at an optimal concentration of 0.15 mmol/L. Importantly, TN-IM outperformed the single-imidazoline ring inhibitor STD-MC under all tested conditions (inhibitor concentration, CO_2_ pressure, temperature, corrosion time, and NaCl concentration), with an inhibition efficiency 1.86% higher than that of STD-MC. Electrochemical measurements demonstrated that TN-IM acts as a mixed-type corrosion inhibitor, with a predominant inhibitory effect on the anodic reaction of N80 steel. The addition of TN-IM significantly reduced the corrosion current density and increased the charge transfer resistance of N80 steel, which is attributed to its adsorption onto the active sites of the steel surface, thereby suppressing the charge transfer process in the electrochemical corrosion reaction. SEM and EDS characterization confirmed that TN-IM forms a dense and stable adsorption film on the N80 steel surface, which effectively mitigates steel corrosion and inhibits the formation of corrosion products. The higher N content on the steel surface in the presence of TN-IM compared with STD-MC indicates a stronger adsorption capacity of TN-IM. This is identified as the key factor underlying its superior corrosion inhibition performance. Mechanistically, the dual-imidazoline ring structure of TN-IM provides multiple adsorption sites for binding to the metal surface, while the tetradecanedioic acid-derived alkyl chain forms a hydrophobic physical barrier. These two features synergistically enhance the corrosion inhibition performance and film-forming stability of the inhibitor. This work provides a new molecular design strategy for the development of high-efficiency imidazoline-based CO_2_ corrosion inhibitors for oil and gas field applications. In future research, we will address the issues related to longer-term corrosion scenarios and conduct comparative EIS or PDP experiments of TN-IM, STD-MC and other new inhibitors. Furthermore, it is necessary to investigate the dynamic corrosion inhibition performance of the newly synthesized inhibitor using an ultra-high-temperature and high-pressure rotating reactor to expand its practical applicability in harsh industrial environments.

## Figures and Tables

**Table 1:** Elemental compositions of N80 (wt%).

Element	C	Si	Mn	P	S	Cu	Ni	Cr	Mo	Fe
Composition	0.450	0.320	1.700	0.014	0.013	0.120	0.040	0.030	0.200	97.113

**Table 2:** Electrochemical parameters from potentiodynamic polarization curves of N80 steel with different concentrations of TN-IM.

Concentration of TN-IM (mmol/L)	*E*_corr_ (V_SCE_)	*Β_a_* (mV·dec^−1^)	*Β_c_* (mV·dec^−1^)	*I_corr_* (mA/cm^−2^)	*η*_2_ (%)
-	−0.781	10.527	−4.010	0.04102	-
0.03	−0.685	25.860	−18.324	0.00789	80.77
0.06	−0.693	25.875	−19.045	0.00600	85.37
0.09	−0.695	28.794	−18.929	0.00569	86.13
0.12	−0.674	27.204	−18.064	0.00518	87.37
0.15	−0.717	22.259	−19.797	0.00512	87.52

**Table 3:** EIS fittings of N80 in different concentrations of TN-IM solutions.

Concentration of TN-IM (mmol/L)	R_s_ (Ω·cm^2^)	CPE_f_		Rf (Ω·cm^2^)	CPE_dl_		R_ct_ (Ω·cm^2^)	η_R_ (%)
		Y_0_ (μΩ^−1^S^n^cm^−2^)	n		Y_0_ (μΩ^−1^S^n^cm^−2^)	n		
-	12.240	-	-	-	278.85	0.74	157.9	-
0.03	10.690	28.41	0.82	38.18	277.18	0.76	991.2	83.64
0.06	11.280	21.15	0.83	41.23	211.53	0.75	1058.0	84.68
0.09	11.540	37.23	0.83	45.42	202.07	0.74	1076.0	85.00
0.12	11.450	30.08	0.84	51.12	123.91	0.76	1169.0	86.19
0.15	11.150	35.85	0.84	58.82	108.88	0.75	1246.0	87.07

## Data Availability

The original contributions presented in this study are included in the article/Supplementary Material. Further inquiries can be directed to the corresponding author.

## Supplementary Materials

The following supporting information can be downloaded at https://www.mdpi.com/article/10.3390/ma19101934/s1, Figure S1. FT-IR spectrum of STD-MC. Figure S2. Optimized structure and Frontal Molecular Orbital Distribution Diagram of TN-IM. Figure S3. Distribution of calculated values for TN-IM-related parameters. Figure S4. Electrostatic potential distribution of TN-IM. Figure S5. Langmuir adsorption isotherms of TN-IM on the surface of N80 at 40°C (a), 50°C (b), 60°C (c), 70°C (d), 80°C (e) and (f) thermodynamic parameter fitting diagram for TN-IM. Figure S6. XPS spectra of N80 steel after corrosion in saturated CO_2_ and 3.5 wt% NaCl solution without (a) and with 0.15 mmol/L TN-IM (b) for 24 h: (a) XPS full spectrum without TN-IM, (b) XPS full spectrum with TN-IM, (c) C1s without TN-IM, (d) C1s with TN-IM, (e) Fe2p without TN-IM, (f) Fe2p with TN-IM, (g) O1s without TN-IM, (h) O1s with TN-IM, (i) N1s with TN-IM. Table S1. Comparative weight loss data for N80 steel protected with TN-IM or STD-MC at different concentrations. Table S2. Comparative weight loss data for N80 steel protected with/without TN-IM or STDMC at different CO_2_ pressures. Table S3. Comparative weight loss data for N80 steel protected with/without TN-IM or STDMC at different corrosion-temperatures. Table S4. Comparative weight loss data for N80 steel protected with/without TN-IM or STDMC at different corrosion times. Table S5. Comparative weight loss data for N80 steel protected with/without TN-IM or STD-MC at different NaCl Concentrations. Table S6. Thermodynamic adsorption parameters of TN-IM on N80. Table S7. The experimental and fitting data obtained from the EIS measurements for N80 added with 0 mmol/L, 0.03 mmol/L, and 0.06 mmol/L of TI-IM. Table S8. The experimental and fitting data obtained from the EIS measurements for N80 added with 0.09 mmol/L, 0.12 mmol/L, and 0.15 mmol/L of TI-IM.
