# Supplementary material for: Synthesis, Characterization, and Corrosion Inhibition Properties of a Novel Quaternary Ammonium Salt Containing Dual-Imidazoline Rings for N80 Carbon Steel Under CO2 Corrosion Conditions
Source: Materials (Basel). 2026 May 8;19(10):1934. doi: 10.3390/ma19101934 (PMC13208163; doi:10.3390/ma19101934)
Supplement: Supplementary file 1 [file materials-19-01934-s001.zip › materials-4179770-supplementary.pdf]

Synthesis, Characterization, and Corrosion Inhibition Properties of a Novel Quaternary Ammonium Salt Containing Dual-Imidazoline Rings for N80 Carbon Steel Under CO_2 Corrosion Conditions

Xiaoping Qin[1] | Xi Chen[1] | Peng Tang[1*] | Cuixia Li[1] | Yangyang Yu[2] | Wei Liu[2] | Guanglin Zhou[3] | Wenzhong Tian[3] | Guangliang Lu[3] Song Qing[1] | Haiyang Tian[1]

[1]School of Chemical Engineering, Sichuan University of Science and Engineering, Zigong 643000, China;
[2]Jidong Oilfield Branch Company, PetroChina Company Limited, Tangshan 063002, China;
[3]Southwest Oil and Gas Branch, China Petroleum and Chemical Corporation, Chengdu 610095, China;
*Correspondence: tpdzyyx@163.com

Figure S1 FT-IR spectrum of STD-MC.

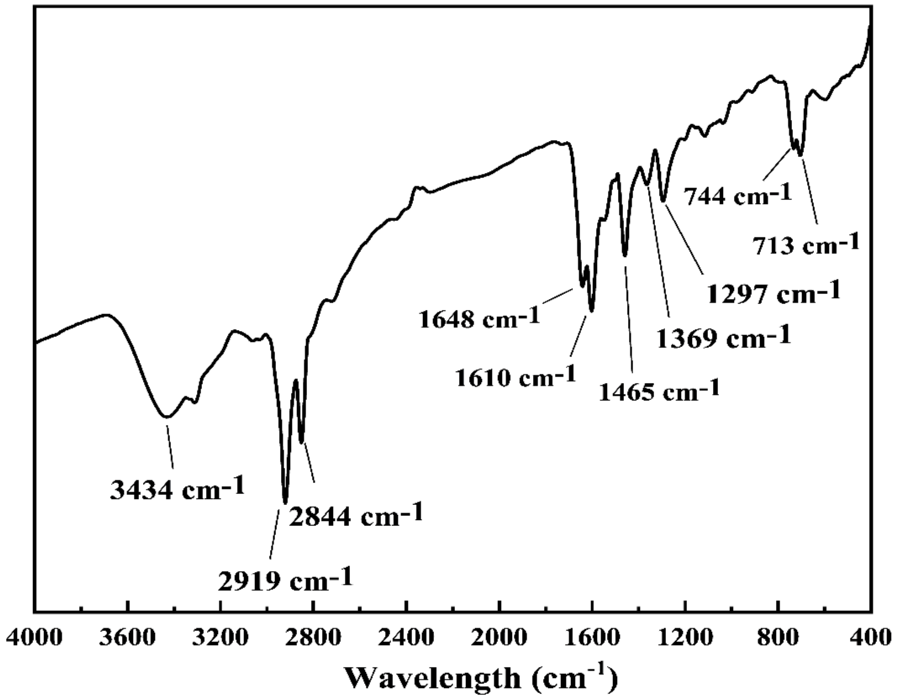

Figure S2 Optimized structure and Frontal Molecular Orbital Distribution Diagram of TN-IM.

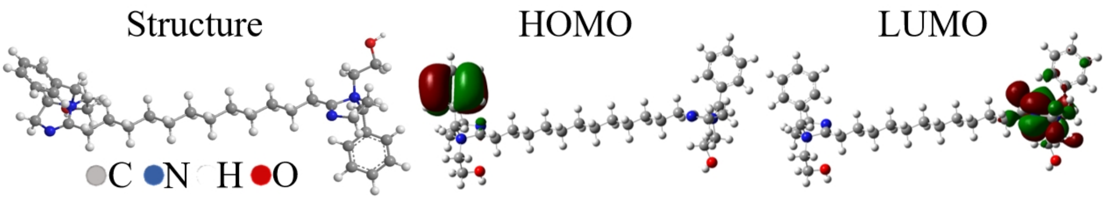

Figure S3 Distribution of calculated values for TN-IM-related parameters.

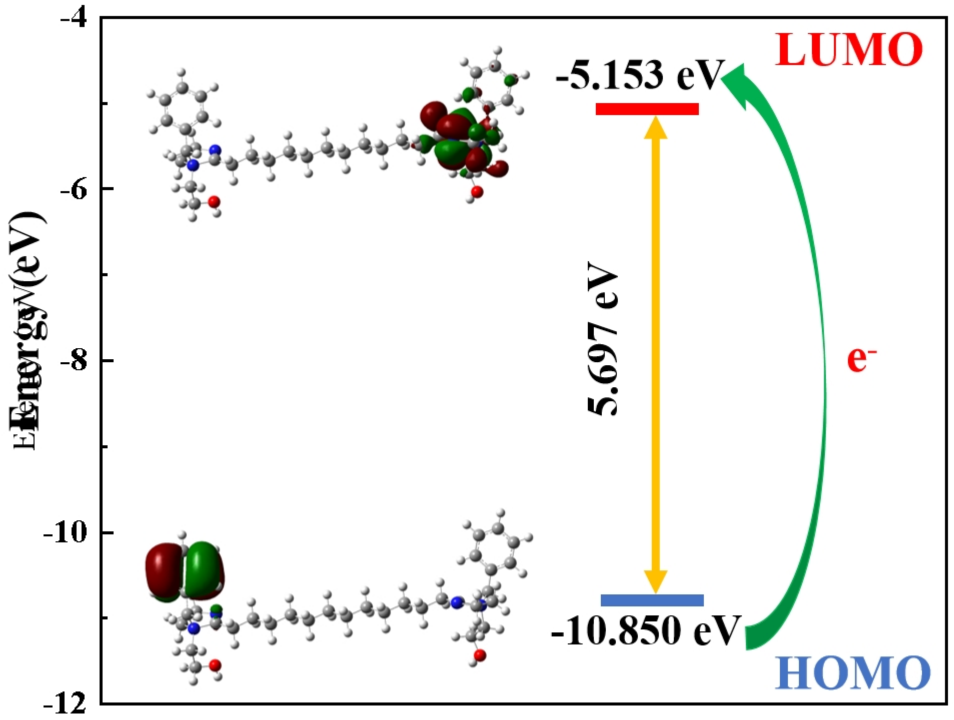

Figure S4 Electrostatic potential distribution of TN-IM.

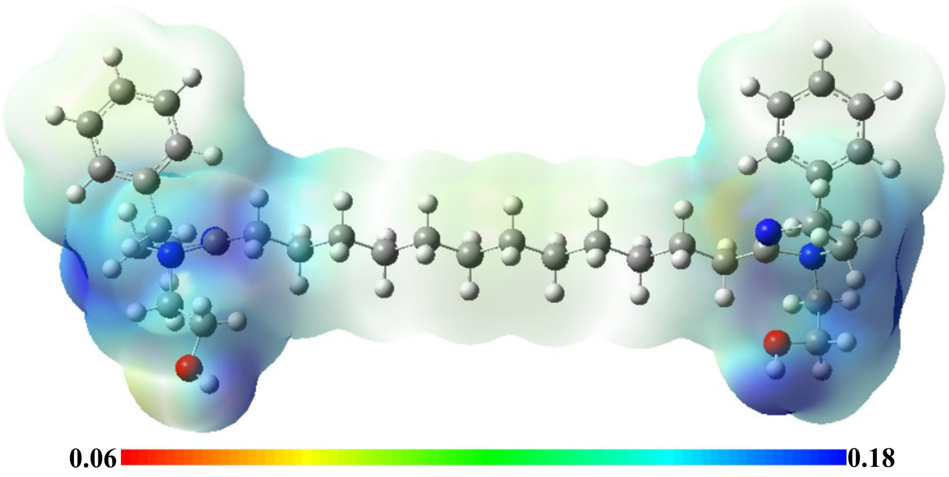

Figure S5 Langmuir adsorption isotherms of TN-IM on the surface of N80 at 40°C (a), 50°C (b), 60°C (c), 70°C (d), 80°C (e) and (f) thermodynamic parameter fitting diagram for TN-IM.

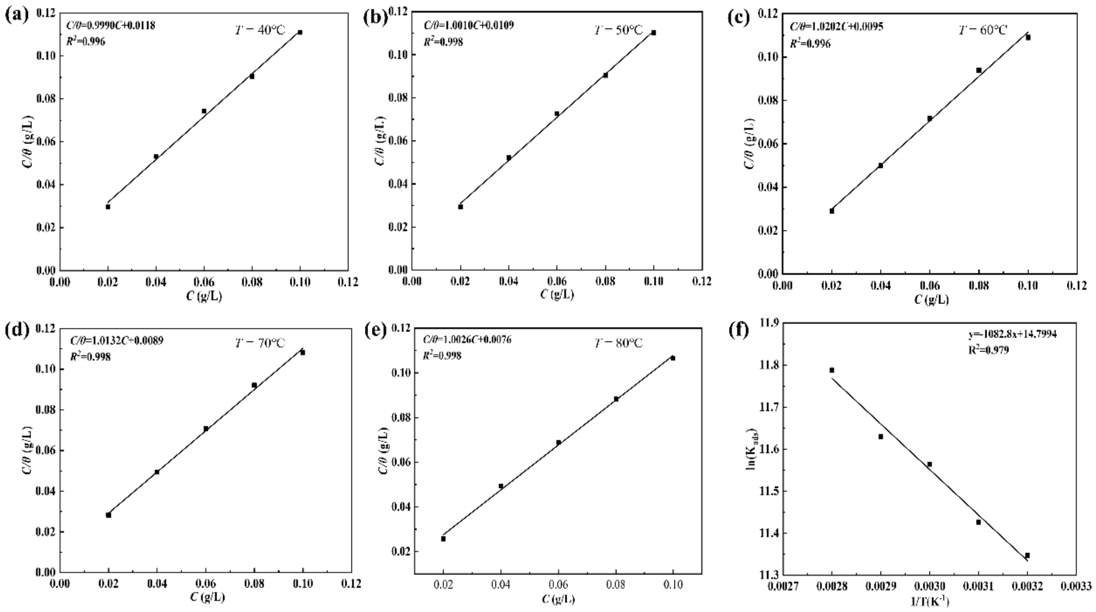

Figure S6 XPS spectra of N80 steel after corrosion in saturated CO_2 and 3.5wt% NaCl solution without (a) and with 0.15 mmol/L TN-IM (b) for 24 h: (a) XPS full spectrum without TN-IM , (b) XPS full spectrum with TN-IM , (c) C1s without TN-IM, (d) C1s with TN-IM, (e) Fe2p without TN-IM, (f) Fe2p with TN-IM, (g) O1s without TN-IM, (h) O1s with TN-IM, (i) N1s with TN-IM

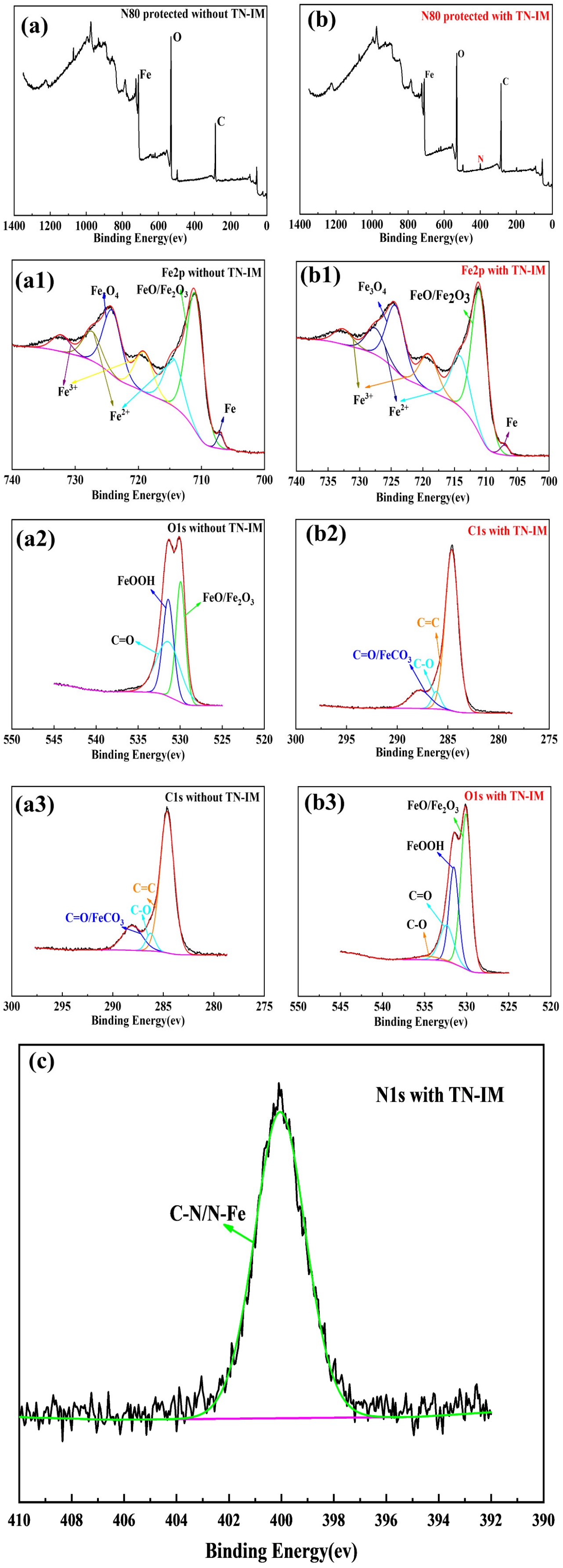
(a)
N80 protected without TN-IM
O
Fe
C
Binding Energy(ev)
(b)
N80 protected with TN-IM
Fe
O
N
C
Binding Energy(ev)
(a1)
Fe2p without TN-IM
Fe3O4
FeO/Fe2O3
Fe3+
Fe2+
Fe
Binding Energy(ev)
(b1)
Fe2p with TN-IM
Fe3O4
FeO/Fe2O3
Fe3+
Fe2+
Fe
Binding Energy(ev)
(a2)
O1s without TN-IM
FeOOH
FeO/Fe2O3
C=O
Binding Energy(ev)
(b2)
C1s with TN-IM
C=C
C=O/FeCO3
C-O
Binding Energy(ev)
(a3)
C1s without TN-IM
C=C
C-O
C=O/FeCO3
Binding Energy(ev)
(b3)
FeO/Fe2O3
O1s with TN-IM
FeOOH
C=O
C-O
Binding Energy(ev)
(c)
N1s with TN-IM
C-N/N-Fe
Binding Energy(ev)

Table S1 Comparative weight loss data for N80 steel protected with TN-IM or STD-MC at different concentrations.

Corrosion inhibitor concentration (mmol/L)	Weight loss (g) of N80 protected with TN-IM	Weight loss (g) of N80 Protected with STD-MC
0.03	$0.0314^{+0.0005}_{-0.0001}$	$0.0075^{+0.0003}_{-0.0002}$
0.06	$0.0207^{+0.0002}_{-0.0004}$	$0.0089^{+0.0005}_{-0.0002}$
0.09	$0.0164^{+0.0003}_{-0.0003}$	$0.0103^{+0.0004}_{-0.0001}$
0.12	$0.0155^{+0.0003}_{-0.0001}$	$0.0179^{+0.0001}_{-0.0003}$
0.15	$0.0084^{+0.0004}_{-0.0002}$	$0.0201^{+0.0004}_{-0.0001}$
0.18	$0.0074^{+0.0004}_{-0.0003}$	$0.0247^{+0.0006}_{-0.0002}$
0.21	$0.0070^{+0.0001}_{-0.0005}$	$0.0388^{+0.0005}_{-0.0005}$

Table S2 Comparative weight loss data for N80 steel protected with/without TN-IM or STD-MC at different CO_2 pressures.

CO_2 pressure (MPa)	Weight loss (g) of N80 without the protection of TN-IM or STD-MC	Weight loss (g) of N80 protected with TN-IM (0.15 mmol/L)	Weight loss (g) of N80 protected with STD-MC (0.15 mmol/L)
0.2	$0.0386^{+0.0002}_{-0.0005}$	$0.0095^{+0.0002}_{-0.0003}$	$0.0138^{+0.0002}_{-0.0006}$
0.4	$0.0755^{+0.0001}_{-0.0004}$	$0.0090^{+0.0005}_{-0.0004}$	$0.0136^{+0.0007}_{-0.0004}$
0.6	$0.1020^{+0.0006}_{-0.0002}$	$0.0084^{+0.0004}_{-0.0002}$	$0.0103^{+0.0004}_{-0.0001}$
0.8	$0.1273^{+0.0004}_{-0.0006}$	$0.0182^{+0.0001}_{-0.0002}$	$0.0227^{+0.0001}_{-0.0005}$
1.0	$0.1520^{+0.0005}_{-0.0001}$	$0.0288^{+0.0004}_{-0.0005}$	$0.0461^{+0.0003}_{-0.0003}$

Table S3 Comparative weight loss data for N80 steel protected with/without TN-IM or STD-MC at different corrosion-temperatures.

Corrosion temperature (°C)	Weight loss (g) of N80 without the protection of TN-IM or STD-MC	Weight loss (g) of N80 protected with TN-IM (0.15 mmol/L)	Weight loss (g) of N80 protected with STD-MC (0.15 mmol/L)
20	$0.0166^{+0.0002}_{-0.0003}$	$0.0043^{+0.0003}_{-0.0001}$	$0.0043^{+0.0006}_{-0.0004}$
40	$0.0616^{+0.0005}_{-0.0006}$	$0.0060^{+0.0002}_{-0.0004}$	$0.0082^{+0.0005}_{-0.0002}$
60	$0.1020^{+0.0002}_{-0.0002}$	$0.0084^{+0.0004}_{-0.0002}$	$0.0103^{+0.0004}_{-0.0001}$
80	$0.1365^{+0.0003}_{-0.0004}$	$0.0205^{+0.0005}_{-0.0003}$	$0.0289^{+0.0006}_{-0.0003}$
100	$0.1540^{+0.0004}_{-0.0005}$	$0.0373^{+0.0001}_{-0.0002}$	$0.0443^{+0.0004}_{-0.0001}$

Table S4 Comparative weight loss data for N80 steel protected with/without TN-IM or STD-MC at different corrosion times.

Corrosion time (h)	Weight loss (g) of N80 without the protection of TN-IM or STD-MC	Weight loss (g) of N80 protected with TN-IM (0.15 mmol/L)	Weight loss (g) of N80 protected with STD-MC (0.15 mmol/L)
6	$0.0459^{+0.0006}_{-0.0004}$	$0.0083^{+0.0003}_{-0.0004}$	$0.0095^{+0.0003}_{-0.0004}$
12	$0.0620^{+0.0004}_{-0.0001}$	$0.0058^{+0.0005}_{-0.0001}$	$0.0086^{+0.0007}_{-0.0005}$
24	$0.1020^{+0.0003}_{-0.0006}$	$0.0084^{+0.0004}_{-0.0002}$	$0.0103^{+0.0004}_{-0.0001}$
36	$0.1424^{+0.0001}_{-0.0006}$	$0.0184^{+0.0002}_{-0.0003}$	$0.0339^{+0.0005}_{-0.0002}$
48	$0.1793^{+0.0004}_{-0.0002}$	$0.0316^{+0.0004}_{-0.0005}$	$0.0590^{+0.0002}_{-0.0006}$

Table S5 Comparative weight loss data for N80 steel protected with/without TN-IM or STD-MC at different NaCl Concentrations.

Concentrations of NaCl (wt%)	Weight loss (g) of N80 without the protection of TN-IM or STD-MC	Weight loss (g) of N80 protected with TN-IM (0.15 mmol/L)	Weight loss (g) of N80 protected with STD-MC (0.15 mmol/L)
3.0	$0.0793^{+0.0006}_{-0.0005}$	$0.0063^{+0.0001}_{-0.0002}$	$0.0070^{+0.0004}_{-0.0004}$
3.5	$0.1020^{+0.0003}_{-0.0001}$	$0.0084^{+0.0004}_{-0.0002}$	$0.0103^{+0.0004}_{-0.0001}$
4.0	$0.1119^{+0.0002}_{-0.0004}$	$0.0117^{+0.0003}_{-0.0004}$	$0.0152^{+0.0002}_{-0.0004}$
4.5	$0.1407^{+0.0005}_{-0.0005}$	$0.0173^{+0.0005}_{-0.0001}$	$0.0230^{+0.0003}_{-0.0001}$
5.0	$0.1721^{+0.0001}_{-0.0003}$	$0.0276^{+0.0002}_{-0.0003}$	$0.0328^{+0.0005}_{-0.0002}$

Table S6 Thermodynamic adsorption parameters of TN-IM on N80

Temperature ℃	K_{ads} L/mol	ΔG^{θ}_{ads} kJ/mol	ΔH^{0}_{ads} kJ/mol	ΔS^{0}_{ads} J/(k·mol)
40	8.4746×10^{4}	-40.0001		
50	9.1743×10^{4}	-41.4906		
60	1.0526×10^{5}	-43.1552	9.0024	156.4344
70	1.1236×10^{5}	-44.6368		
80	1.3158×10^{5}	-46.4012		

Table S7 The experimental and fitting data obtained from the EIS measurements for N80 added with 0 mmol/L, 0.03 mmol/L, and 0.06 mmol/L of TI-IM

0 mmol/L of TI-IM				0.03 mmol/L of TI-IM				0.06 mmol/L of TI-IM			
Experimental data		Fitting data		Experimental data		Fitting data		Experimental data		Fitting data	
Z'/ohm	Z"/ohm	Z'/ohm	Z"/ohm	Z'/ohm	Z"/ohm	Z'/ohm	Z"/ohm	Z'/ohm	Z"/ohm	Z'/ohm	Z"/ohm
12.35	-0.307	12.35	-0.31	10.74	-0.32	10.74	-0.3244	5.29	0.65	5.29	-0.03574
12.36	-0.3065	12.36	-0.31	10.75	-0.31	10.75	-0.307	5.24	0.52	5.24	-0.04435
12.37	-0.3226	12.37	-0.32	10.76	-0.31	10.76	-0.3145	5.21	0.41	5.21	-0.05296
12.38	-0.3465	12.38	-0.35	10.76	-0.31	10.76	-0.3107	5.18	0.30	5.18	-0.06157
12.41	-0.3785	12.41	-0.38	10.78	-0.32	10.78	-0.322	5.16	0.20	5.16	-0.07018
12.43	-0.4073	12.43	-0.41	10.80	-0.34	10.80	-0.3393	5.15	0.10	5.15	-0.07879
12.46	-0.4419	12.46	-0.44	10.82	-0.36	10.82	-0.3592	5.14	0.00	5.14	-0.0874
12.48	-0.4961	12.48	-0.50	10.84	-0.40	10.84	-0.395	5.14	-0.10	5.14	-0.09601
12.51	-0.5608	12.51	-0.56	10.86	-0.44	10.86	-0.4431	5.16	-0.20	5.16	-0.1987
12.55	-0.6291	12.55	-0.63	10.88	-0.49	10.88	-0.49	5.17	-0.30	5.17	-0.3046
12.59	-0.7132	12.59	-0.71	10.92	-0.55	10.92	-0.5513	5.21	-0.41	5.21	-0.4137
12.64	-0.8182	12.64	-0.82	10.95	-0.63	10.95	-0.6287	5.24	-0.53	5.24	-0.5308
12.69	-0.9335	12.69	-0.93	10.99	-0.71	10.99	-0.7118	5.29	-0.66	5.29	-0.6558
12.75	-1.067	12.75	-1.07	11.04	-0.81	11.04	-0.814	5.35	-0.79	5.35	-0.788
12.82	-1.226	12.82	-1.23	11.10	-0.93	11.10	-0.9316	5.42	-0.93	5.42	-0.9335
12.91	-1.409	12.91	-1.41	11.16	-1.07	11.16	-1.068	5.50	-1.09	5.50	-1.093
13.00	-1.614	13.00	-1.61	11.24	-1.22	11.24	-1.22	5.58	-1.27	5.58	-1.266
13.11	-1.857	13.11	-1.86	11.33	-1.40	11.33	-1.399	5.69	-1.46	5.69	-1.458
13.23	-2.135	13.23	-2.14	11.41	-1.60	11.41	-1.601	5.80	-1.68	5.80	-1.676
13.37	-2.45	13.37	-2.45	11.52	-1.84	11.52	-1.839	5.92	-1.92	5.92	-1.917
13.53	-2.821	13.53	-2.82	11.64	-2.12	11.64	-2.116	6.06	-2.20	6.06	-2.195
13.71	-3.249	13.71	-3.25	11.78	-2.43	11.78	-2.429	6.21	-2.52	6.21	-2.516
13.92	-3.746	13.92	-3.75	11.93	-2.80	11.93	-2.8	6.38	-2.87	6.38	-2.873
14.16	-4.322	14.16	-4.32	12.10	-3.23	12.10	-3.23	6.57	-3.30	6.57	-3.301
14.44	-5.007	14.44	-5.01	12.30	-3.72	12.30	-3.722	6.77	-3.79	6.77	-3.79
14.77	-5.699	14.77	-5.70	12.52	-4.22	12.52	-4.221	7.01	-4.30	7.01	-4.297

15.14	-6.603	15.14	-6.60	12.77	-4.89	12.77	-4.893	7.27	-4.96	7.27	-4.962
15.60	-7.69	15.60	-7.69	13.08	-5.71	13.08	-5.708	7.59	-5.77	7.59	-5.772
16.13	-8.906	16.13	-8.91	13.42	-6.63	13.42	-6.625	7.94	-6.69	7.94	-6.688
16.78	-10.35	16.78	-10.35	13.83	-7.73	13.83	-7.729	8.36	-7.79	8.36	-7.786
17.50	-11.87	17.50	-11.87	14.27	-8.92	14.27	-8.917	8.82	-8.97	8.82	-8.969
18.43	-13.82	18.43	-13.82	14.84	-10.46	14.84	-10.46	9.41	-10.50	9.41	-10.5
19.44	-15.85	19.44	-15.85	15.45	-12.10	15.45	-12.1	10.05	-12.13	10.05	-12.13
20.74	-18.36	20.74	-18.36	16.23	-14.16	16.23	-14.16	10.88	-14.18	10.88	-14.18
22.10	-20.93	22.10	-20.93	17.05	-16.34	17.05	-16.34	11.75	-16.31	11.75	-16.31
23.70	-23.85	23.70	-23.85	18.02	-18.85	18.02	-18.85	12.78	-18.77	12.78	-18.77
25.98	-27.88	25.98	-27.88	19.43	-22.43	19.43	-22.43	14.27	-22.25	14.27	-22.25
28.36	-31.09	28.36	-31.09	20.99	-25.39	20.99	-25.39	15.87	-25.14	15.87	-25.14
31.15	-35.52	31.15	-35.52	22.72	-29.57	22.72	-29.57	17.67	-29.15	17.67	-29.15
34.74	-41.26	34.74	-41.26	25.10	-35.09	25.10	-35.09	20.13	-34.40	20.13	-34.4
37.91	-46.14	37.91	-46.14	27.35	-39.79	27.35	-39.79	22.37	-38.87	22.37	-38.87
42.23	-52.49	42.23	-52.49	30.36	-46.16	30.36	-46.16	25.40	-44.89	25.40	-44.89
47.24	-59.52	47.24	-59.52	34.02	-53.40	34.02	-53.4	28.98	-51.71	28.98	-51.71
53.07	-67.32	53.07	-67.32	38.41	-61.61	38.41	-61.61	33.20	-59.45	33.20	-59.45
59.86	-75.93	59.86	-75.93	43.75	-70.90	43.75	-70.9	38.25	-68.23	38.25	-68.23
67.81	-85.39	67.81	-85.39	50.20	-81.33	50.20	-81.33	44.22	-78.07	44.22	-78.07
77.09	-95.81	77.09	-95.81	58.01	-93.01	58.01	-93.01	51.31	-89.24	51.31	-89.24
87.91	-107.1	87.91	-107.10	67.40	-105.90	67.40	-105.9	59.77	-101.80	59.77	-101.8
100.50	-119	100.50	-119.00	78.74	-120.10	78.74	-120.1	69.71	-115.50	69.71	-115.5
115.00	-132	115.00	-132.00	92.04	-135.40	92.04	-135.4	81.56	-131.10	81.56	-131.1
131.90	-145.2	131.90	-145.20	107.90	-151.80	107.90	-151.8	95.55	-147.90	95.55	-147.9
151.30	-158.8	151.30	-158.80	126.50	-168.80	126.50	-168.8	112.00	-166.30	112.00	-166.3
173.80	-172.1	173.80	-172.10	148.60	-186.40	148.60	-186.4	131.70	-185.90	131.70	-185.9
199.00	-185.4	199.00	-185.40	173.60	-204.40	173.60	-204.4	154.10	-207.20	154.10	-207.2
227.60	-197.8	227.60	-197.80	202.30	-222.90	202.30	-222.9	180.70	-230.30	180.70	-230.3
258.00	-207.2	258.00	-207.20	233.50	-238.80	233.50	-238.8	210.50	-252.10	210.50	-252.1
293.30	-216	293.30	-216.00	270.10	-256.30	270.10	-256.3	246.40	-277.60	246.40	-277.6
327.70	-219.3	327.70	-219.30	307.40	-268.60	307.40	-268.6	284.90	-298.80	284.90	-298.8
371.90	-222.6	371.90	-222.60	356.20	-287.10	356.20	-287.1	335.80	-329.50	335.80	-329.5
407.10	-216.8	407.10	-216.80	398.30	-293.40	398.30	-293.4	383.90	-346.40	383.90	-346.4
440.20	-206.5	440.20	-206.50	441.20	-296.10	441.20	-296.1	436.10	-359.60	436.10	-359.6
474.00	-190.8	474.00	-190.80	488.70	-294.20	488.70	-294.2	494.40	-366.70	494.40	-366.7
499.30	-174.1	499.30	-174.10	526.20	-287.80	526.20	-287.8	547.70	-368.20	547.70	-368.2
522.70	-155.8	522.70	-155.80	566.30	-281.00	566.30	-281	604.30	-366.80	604.30	-366.8
542.20	-136.3	542.20	-136.30	605.60	-271.50	605.60	-271.5	660.60	-359.20	660.60	-359.2
557.90	-116.9	557.90	-116.90	641.30	-259.10	641.30	-259.1	715.50	-346.20	715.50	-346.2
569.40	-97.83	569.40	-97.83	675.80	-243.80	675.80	-243.8	768.50	-328.70	768.50	-328.7
578.40	-80.37	578.40	-80.37	706.20	-228.80	706.20	-228.8	819.70	-307.70	819.70	-307.7
584.10	-65.01	584.10	-65.01	735.60	-212.50	735.60	-212.5	865.50	-281.80	865.50	-281.8
587.70	-51.92	587.70	-51.92	763.20	-194.60	763.20	-194.6	905.40	-253.20	905.40	-253.2
589.40	-41.12	589.40	-41.12	783.40	-175.80	783.40	-175.8	941.00	-223.00	941.00	-223
590.80	-33.64	590.80	-33.64	802.60	-156.10	802.60	-156.1	970.20	-191.70	970.20	-191.7
592.90	-29.23	592.90	-29.23	815.30	-135.80	815.30	-135.8	993.40	-162.40	993.40	-162.4

Table S8 The experimental and fitting data obtained from the EIS measurements for N80 added with 0.09 mmol/L, 0.12 mmol/L, and 0.15 mmol/L of TI-IM

0.09 mmol/L of TI-IM				0.12 mmol/L of TI-IM				0.15 mmol/L of TI-IM			
Experimental data		Fitting data		Experimental data		Fitting data		Experimental data		Fitting data	
4.42	0.58	4.42	0	8.63	-0.06	8.63	-0.05874	11.15	-0.18	11.15	-0.1771
4.39	0.43	4.39	0	8.63	-0.10	8.63	-0.09607	11.15	-0.19	11.15	-0.1942
4.39	0.30	4.39	-0.00019	8.62	-0.14	8.62	-0.1358	11.16	-0.24	11.16	-0.2352
4.39	0.18	4.39	-0.0073	8.63	-0.18	8.63	-0.1805	11.17	-0.25	11.17	-0.2524
4.40	0.07	4.40	-0.01441	8.65	-0.23	8.65	-0.2291	11.19	-0.29	11.19	-0.2889
4.42	-0.02	4.42	-0.02152	8.67	-0.28	8.67	-0.2791	11.22	-0.32	11.22	-0.3217
4.44	-0.11	4.44	-0.1114	8.68	-0.33	8.68	-0.33	11.24	-0.36	11.24	-0.3593
4.46	-0.20	4.46	-0.1979	8.71	-0.39	8.71	-0.39	11.27	-0.41	11.27	-0.4064
4.49	-0.28	4.49	-0.2828	8.73	-0.46	8.73	-0.4607	11.30	-0.46	11.30	-0.4617
4.53	-0.37	4.53	-0.3682	8.77	-0.54	8.77	-0.5375	11.33	-0.52	11.33	-0.523
4.56	-0.46	4.56	-0.458	8.81	-0.62	8.81	-0.624	11.38	-0.59	11.38	-0.5928
4.60	-0.55	4.60	-0.5512	8.85	-0.72	8.85	-0.7244	11.42	-0.67	11.42	-0.6749
4.65	-0.65	4.65	-0.6541	8.91	-0.83	8.91	-0.8344	11.47	-0.76	11.47	-0.7628

4.70	-0.77	4.70	-0.7688	8.98	-0.96	8.98	-0.9574	11.53	-0.87	11.53	-0.8658
4.76	-0.90	4.76	-0.8964	9.06	-1.10	9.06	-1.1	11.60	-0.98	11.60	-0.984
4.82	-1.04	4.82	-1.04	9.14	-1.26	9.14	-1.26	11.67	-1.12	11.67	-1.121
4.89	-1.20	4.89	-1.204	9.23	-1.44	9.23	-1.44	11.75	-1.28	11.75	-1.276
4.97	-1.39	4.97	-1.393	9.34	-1.64	9.34	-1.643	11.84	-1.46	11.84	-1.456
5.06	-1.61	5.06	-1.61	9.46	-1.88	9.46	-1.877	11.94	-1.66	11.94	-1.662
5.17	-1.86	5.17	-1.861	9.60	-2.14	9.60	-2.142	12.05	-1.90	12.05	-1.896
5.28	-2.15	5.28	-2.151	9.75	-2.45	9.75	-2.449	12.17	-2.18	12.17	-2.175
5.42	-2.49	5.42	-2.488	9.92	-2.81	9.92	-2.808	12.32	-2.49	12.32	-2.49
5.57	-2.88	5.57	-2.875	10.11	-3.21	10.11	-3.205	12.47	-2.86	12.47	-2.862
5.74	-3.33	5.74	-3.331	10.32	-3.68	10.32	-3.675	12.64	-3.30	12.64	-3.295
5.95	-3.86	5.95	-3.856	10.55	-4.22	10.55	-4.22	12.86	-3.80	12.86	-3.799
6.19	-4.41	6.19	-4.408	10.83	-4.77	10.83	-4.774	13.09	-4.30	13.09	-4.302
6.45	-5.12	6.45	-5.117	11.13	-5.50	11.13	-5.503	13.36	-4.99	13.36	-4.985
6.77	-5.98	6.77	-5.976	11.50	-6.39	11.50	-6.389	13.69	-5.81	13.69	-5.811
7.14	-6.94	7.14	-6.943	11.91	-7.38	11.91	-7.383	14.07	-6.73	14.07	-6.734
7.59	-8.10	7.59	-8.1	12.40	-8.57	12.40	-8.57	14.52	-7.84	14.52	-7.841
8.07	-9.34	8.07	-9.339	12.92	-9.84	12.92	-9.843	15.02	-9.02	15.02	-9.018
8.70	-10.93	8.70	-10.93	13.60	-11.48	13.60	-11.48	15.66	-10.53	15.66	-10.53
9.37	-12.63	9.37	-12.63	14.33	-13.22	14.33	-13.22	16.35	-12.12	16.35	-12.12
10.23	-14.74	10.23	-14.74	15.25	-15.40	15.25	-15.4	17.24	-14.11	17.24	-14.11
11.14	-16.95	11.14	-16.95	16.21	-17.66	16.21	-17.66	18.18	-16.16	18.18	-16.16
12.21	-19.48	12.21	-19.48	17.33	-20.26	17.33	-20.26	19.26	-18.51	19.26	-18.51
13.74	-23.07	13.74	-23.07	18.95	-23.95	18.95	-23.95	20.81	-21.81	20.81	-21.81
15.37	-26.05	15.37	-26.05	20.68	-26.97	20.68	-26.97	22.47	-24.49	22.47	-24.49
17.20	-30.18	17.20	-30.18	22.60	-31.23	22.60	-31.23	24.30	-28.27	24.30	-28.27
19.66	-35.62	19.66	-35.62	25.19	-36.81	25.19	-36.81	26.77	-33.21	26.77	-33.21
21.92	-40.25	21.92	-40.25	27.57	-41.55	27.57	-41.55	29.02	-37.39	29.02	-37.39
24.95	-46.51	24.95	-46.51	30.76	-47.96	30.76	-47.96	31.98	-43.01	31.98	-43.01
28.52	-53.62	28.52	-53.62	34.53	-55.23	34.53	-55.23	35.47	-49.36	35.47	-49.36
32.76	-61.72	32.76	-61.72	38.97	-63.47	38.97	-63.47	39.55	-56.55	39.55	-56.55
37.83	-70.91	37.83	-70.91	44.29	-72.86	44.29	-72.86	44.36	-64.68	44.36	-64.68
43.82	-81.32	43.82	-81.32	50.60	-83.38	50.60	-83.38	50.03	-73.81	50.03	-73.81
51.08	-93.13	51.08	-93.13	58.12	-95.29	58.12	-95.29	56.71	-84.16	56.71	-84.16
59.62	-106.30	59.62	-106.3	67.13	-108.60	67.13	-108.6	64.55	-95.66	64.55	-95.66
69.74	-121.20	69.74	-121.2	77.78	-123.10	77.78	-123.1	73.79	-108.50	73.79	-108.5
81.97	-137.40	81.97	-137.4	90.44	-139.60	90.44	-139.6	84.57	-122.70	84.57	-122.7
96.33	-155.20	96.33	-155.2	105.40	-157.30	105.40	-157.3	97.16	-138.30	97.16	-138.3
113.30	-174.60	113.30	-174.6	123.00	-176.30	123.00	-176.3	111.80	-155.30	111.80	-155.3
133.70	-195.30	133.70	-195.3	144.00	-196.60	144.00	-196.6	128.90	-174.00	128.90	-174
157.00	-217.80	157.00	-217.8	168.00	-218.20	168.00	-218.2	149.20	-194.20	149.20	-194.2
184.30	-242.20	184.30	-242.2	196.00	-241.50	196.00	-241.5	172.10	-216.40	172.10	-216.4
215.00	-265.00	215.00	-265	227.20	-263.00	227.20	-263	197.90	-238.00	197.90	-238
251.70	-291.70	251.70	-291.7	264.20	-287.90	264.20	-287.9	228.90	-264.20	228.90	-264.2
291.00	-313.90	291.00	-313.9	303.30	-308.20	303.30	-308.2	262.00	-287.20	262.00	-287.2
342.50	-346.60	342.50	-346.6	354.90	-338.30	354.90	-338.3	306.90	-322.40	306.90	-322.4
390.90	-365.40	390.90	-365.4	402.40	-354.60	402.40	-354.6	349.00	-345.40	349.00	-345.4
442.40	-381.40	442.40	-381.4	452.30	-367.90	452.30	-367.9	394.80	-366.30	394.80	-366.3
502.20	-393.70	502.20	-393.7	509.10	-377.20	509.10	-377.2	449.70	-386.30	449.70	-386.3
553.10	-398.10	553.10	-398.1	556.30	-379.30	556.30	-379.3	497.70	-398.20	497.70	-398.2
607.70	-401.50	607.70	-401.5	608.10	-381.70	608.10	-381.7	552.90	-412.50	552.90	-412.5
663.70	-401.90	663.70	-401.9	659.30	-381.50	659.30	-381.5	609.80	-422.70	609.80	-422.7
718.50	-398.50	718.50	-398.5	710.10	-378.50	710.10	-378.5	670.50	-430.40	670.50	-430.4
772.40	-391.00	772.40	-391	759.10	-372.70	759.10	-372.7	731.70	-433.60	731.70	-433.6
825.10	-380.00	825.10	-380	808.10	-365.30	808.10	-365.3	794.60	-432.60	794.60	-432.6
874.90	-364.70	874.90	-364.7	854.50	-355.90	854.50	-355.9	857.80	-426.70	857.80	-426.7
921.00	-345.90	921.00	-345.9	899.30	-344.70	899.30	-344.7	921.70	-416.30	921.70	-416.3
964.30	-323.50	964.30	-323.5	942.00	-331.70	942.00	-331.7	984.10	-400.30	984.10	-400.3
1005.00	-297.50	1005.00	-297.5	981.40	-316.30	981.40	-316.3	1043.00	-378.40	1043.00	-378.4
1043.00	-270.00	1043.00	-270	1019.00	-299.40	1019.00	-299.4	1097.00	-350.80	1097.00	-350.8
